# Venom mass fingerprints for species delimitation, reloaded: A test case with Italian scorpions (*Euscorpius*)

**DOI:** 10.1016/j.toxcx.2026.100265

**Published:** 2026-09-21

**Authors:** Javier Blasco-Aróstegui, Massimiliano Roppo, Lorenzo Prendini, Reinhard Predel

**Affiliations:** a Centre for Ecology, Evolution and Environmental Changes, Departamento de Biologia Animal, Faculdade de Ciências, Universidade de Lisboa, Campo Grande 016, Lisbon, 1749-016, Portugal; b Arachnology Lab and Scorpion Systematics Research Group, Division of Invertebrate Zoology, American Museum of Natural History, New York, United States; c Institute of Zoology, University of Cologne, Zülpicher Str. 47b, Köln, 50674, Germany

**Keywords:** Venomics, Chemotaxonomy, DNA barcoding, Hierarchical clustering, Phenetics, Cryptic diversity

## Abstract

Scorpion venoms are complex mixtures of bioactive compounds including proteins, peptides and enzymes. Although venoms exhibit substantial intraspecific variation, some compounds appear to be species-specific, highlighting their potential as chemotaxonomic markers. Venom mass fingerprints (MFPs) derived from Matrix-Assisted Laser Desorption/Ionization Time-of-Flight (MALDI-TOF) Mass Spectrometry (MS) have been proposed as a means of delimiting species. However, their utility for species delimitation has rarely been evaluated against independent sources of taxonomic evidence. In the present study, the performance of MALDI-TOF MS-based venom peptide barcoding (mass ranges of *m/z* 800‒4500 and *m/z* 3000‒10,000) was assessed in 52 populations of eight Italian species of the scorpion genus *Euscorpius* Thorell, 1876, representing different levels of evolutionary divergence, by comparing venom MFPs with mitochondrial Cytochrome *c* Oxidase Subunit I (COI) DNA barcodes. Non-metric multidimensional scaling (NMDS) based on Jaccard distances of 52 venom MFPs (one per population) recovered seven distinct, statistically supported species clusters that were mostly congruent with analyses based on COI. Hierarchical clustering analyses using the intersection-based distances of the same MFPs consistently recovered the two major *Euscorpius* species complexes and revealed geographically structured subclades congruent with COI data. Venom profiles successfully discriminated among both closely and distantly related species in similarity-based analyses, recovering patterns broadly consistent with established phylogenetic hypotheses. Phylogenetic reconstruction with Maximum Likelihood, based solely on venom characters, obtained less resolution at deeper evolutionary levels, with lower nodal support and some incongruence with the topology obtained from the DNA sequences. The results demonstrate that venom MFPs constitute a useful source of data for species delimitation, particularly for resolving cryptic species complexes and morphologically conserved taxa, complementing morphological and DNA sequence data in integrative taxonomy. Broader taxon sampling, together with improved signal detection and character selection (filtering ion signals), may increase the utility of venom MFPs for phylogenetic analysis by increasing the number of phylogenetically informative characters.

## Highlights

• The first rigorous comparison of venom profiles with DNA barcoding and morphology in a monophyletic scorpion group.
• Venom mass fingerprints successfully discriminated both closely and distantly related *Euscorpius* species.
• NMDS and hierarchical clustering analyses recovered species clusters largely congruent with mitochondrial COI.
• Venom-based phylogenetic inference and similarity analyses showed lower resolution than DNA-based analyses.
• Venom peptide barcoding provides a rapid complementary tool for integrative species delimitation.

## Graphical abstract

**Figure:**
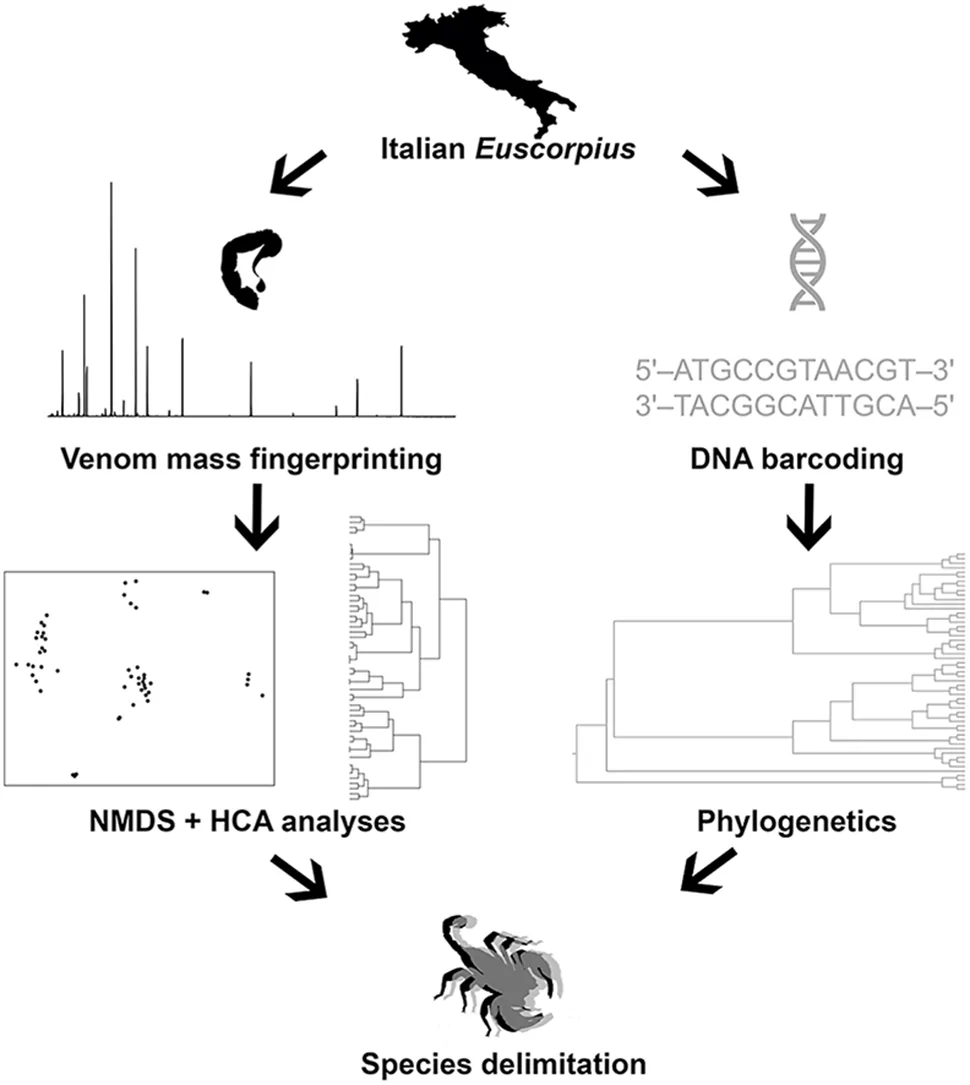

## 1 Introduction

Scorpion venoms are complex, highly plastic ‘cocktails’ of bioactive compounds (Watt and Simard, 1984; De la Vega et al., 2010; Morgenstern and King, 2013; Mille et al., 2014) including short-to medium-sized peptides and high molecular weight proteins that serve various functions, e.g., neurotoxicity, cytotoxicity, antimicrobial activity, and/or enzymatic processes (Possani et al., 2000; Batista et al., 2004; De la Vega and Possani, 2004; Ramírez-Carreto et al., 2012; Murdoch et al., 2026). Unlike their conserved body-plan (Dunlop et al., 2008), the venom of scorpions evolved dynamically under diverse selection pressures, giving rise to specialized toxin arsenals (Zhijian et al., 2006; De la Vega et al., 2010). Scorpion venom composition is known to vary intraspecifically in relation to geography, diet, sex, developmental stage and ancestry (Dyason et al., 2002; Abdel-Rahman et al., 2009; Pucca et al., 2014; Schaffrath et al., 2018; Tobassum et al., 2018; Olguín-Pérez et al., 2021; Krämer et al., 2022; Ajdi et al., 2024, 2025; El Joud et al., 2024; Reitsema-Wang et al., 2025). Nevertheless, several venom components appear to be stable and consistently expressed at the species level even in allopatric populations (Schaffrath et al., 2018; Ajdi et al., 2024).

The potential utility of venom in scorpion systematics was identified early on by Goyffon and Kovoor (1978), based on a broad compositional distinction between the venoms of buthoid (i.e., Buthidae Koch, 1837a) and non-buthoid (i.e., Bothriuridae Simon, 1880; Chactidae Pocock, 1893; Diplocentridae Karsch, 1880; Scorpionidae Latreille, 1802 and Vaejovidae Thorell, 1876) taxa. Although this idea stimulated subsequent investigation, its broader implications remained largely unexplored for two decades, until the first studies of venom “barcodes” or mass fingerprints (MFPs) were presented using Matrix Assisted Laser Desorption/Ionization Time of Flight (MALDI-TOF) mass spectrometry (MS) (Debont et al., 1998; Dyason et al., 2002; Schwartz et al., 2008; Schaffrath and Predel, 2014). MALDI-TOF MS is well-suited for the analysis of scorpion venoms due to its speed, high sensitivity, and analytical accuracy (James, 2001). The resulting mass spectra yield characteristic, individual-specific profiles (i.e., MFPs) that offer informative chemotaxonomic markers, provided they contain sufficient diagnostic signal, i.e., that interspecific variation consistently exceeds intraspecific variation. Venom profiles may be treated as multivariate characters suitable for quantitative comparison, clustering, and hypothesis testing in phenetic or numerical taxonomy frameworks (Sokal and Sneath, 1963; Schaffrath, 2017; Schaffrath et al., 2018; Ajdi et al., 2024). As with morphological characters, the analysis begins by compiling a data matrix in which each operational taxonomic unit (OTU)—whether an individual, population, or species—is represented by a vector of peptide masses derived from ion signals in MFPs. This matrix underpins proximity analyses, in which pairwise similarity or dissimilarity among OTUs is quantified using appropriate distance measures (Bock, 2005; Carugo, 2010), forming the basis for clustering or ordination (Xu and Tian, 2015; Schaffrath, 2017). For simplicity and robustness, MFP data are often reduced to binary form, coding peptide presence and absence. This facilitates the use of classical similarity coefficients while minimizing the influence of intensity variation. Consistent with their genomic similarity, individuals from the same population are expected to exhibit broadly similar venom compositions, yielding comparable “venomes” *sensu* Pimenta et al. (2003).

Several challenges remain in MS-based venomics, however. These include inaccuracies in the automatic detection and assignment of monoisotopic peptide and protein signals, sampling bias at both individual and population levels, limited reproducibility across instruments and laboratories, difficulties in data normalization and cross-study comparability, and the slow, labor-intensive process of scoring the presence and absence of species-specific peaks into a matrix (Schaffrath and Predel, 2014; Schaffrath, 2017; Schaffrath et al., 2018; Ajdi et al., 2025). Consequently, the taxonomic resolution achievable using venom MFPs alone remains uncertain and their broad utility for species delimitation has yet to be demonstrated by comparison with other sources of data (e.g., morphology and DNA).

The scorpion genus *Euscorpius* Thorell, 1876 of the family Euscorpiidae Laurie, 1896, is a monophyletic group, comprising approximately 74 species distributed across the Western Palearctic, in diverse habitats and often great abundance (Gantenbein et al., 1999; Parmakelis et al., 2013; Podnar et al., 2021; Blasco-Aróstegui and Prendini, 2023, 2024). These characteristics, together with the ease with which they may be collected, make these scorpions an excellent model system for testing venom-based species delimitation. The Italian Peninsula represents an ideal study area, as its complex topography promoted diversification and provided refugia during unfavorable climatic oscillations (Stefani et al., 2012), resulting in a rich *Euscorpius* fauna that includes well-defined species and complexes of morphologically similar, cryptic species (Gantenbein et al., 1999; Fet et al., 2003; Salomone et al., 2007; Parmakelis et al., 2013; Tropea, 2017; Podnar et al., 2021; Tropea and Parmakelis, 2022; Blasco‐Aróstegui et al., 2026).

The aim of the present study was to assess the utility of venom-based peptide barcoding for species delimitation by comparing venom MFPs with mitochondrial Cytochrome Oxidase *c* Subunit I (COI) DNA barcodes from the same populations of morphologically delimited Italian *Euscorpius* species, to evaluate whether venom MFPs can serve both as an independent source of taxonomic evidence and a reliable identification tool, particularly for taxa characterized by high levels of morphological conservatism and cryptic diversity, such as scorpions. Specific aims were to compare MFPs and DNA barcodes (1) among closely related species of the same complex, e.g., *Euscorpius concinnus* (Koch, 1837b) and *Euscorpius sicanus* (Koch, 1837b) ; (2) among divergent species of the same lineage, e.g., *Euscorpius celanus* Tropea, 2012 vs. *Euscorpius garganicus* Caporiacco, 1950; and (3) among representatives of different lineages, e.g., *Euscorpius aquilejensis* (Koch, 1837b) vs. *Euscorpius italicus* (Herbst, 1800).

## 2 Material and methods

### 2.1 Taxon and population sampling

Fieldwork was conducted across the Italian Peninsula, targeting historical localities of eight species of *Euscorpius*: *E. concinnus*, *E. latinus* Tropea and Parmakelis, 2022, and *E. trejaensis* Tropea and Parmakelis, 2022, representing the *concinnus* complex; *E. celanus*, *E. garganicus* and *E. salentinus* Tropea, 2017, representing the *sicanus* complex; and the divergent species *E. aquilejensis* and *E. italicus* (Fig. 1, Fig. 2). The basal-most European euscorpiid *Tetratrichobothrius flavicaudis* (DeGeer, 1778) was included as outgroup (Parmakelis et al., 2013; Podnar et al., 2021; Blasco‐Aróstegui et al., 2026).

**Fig. 1:**
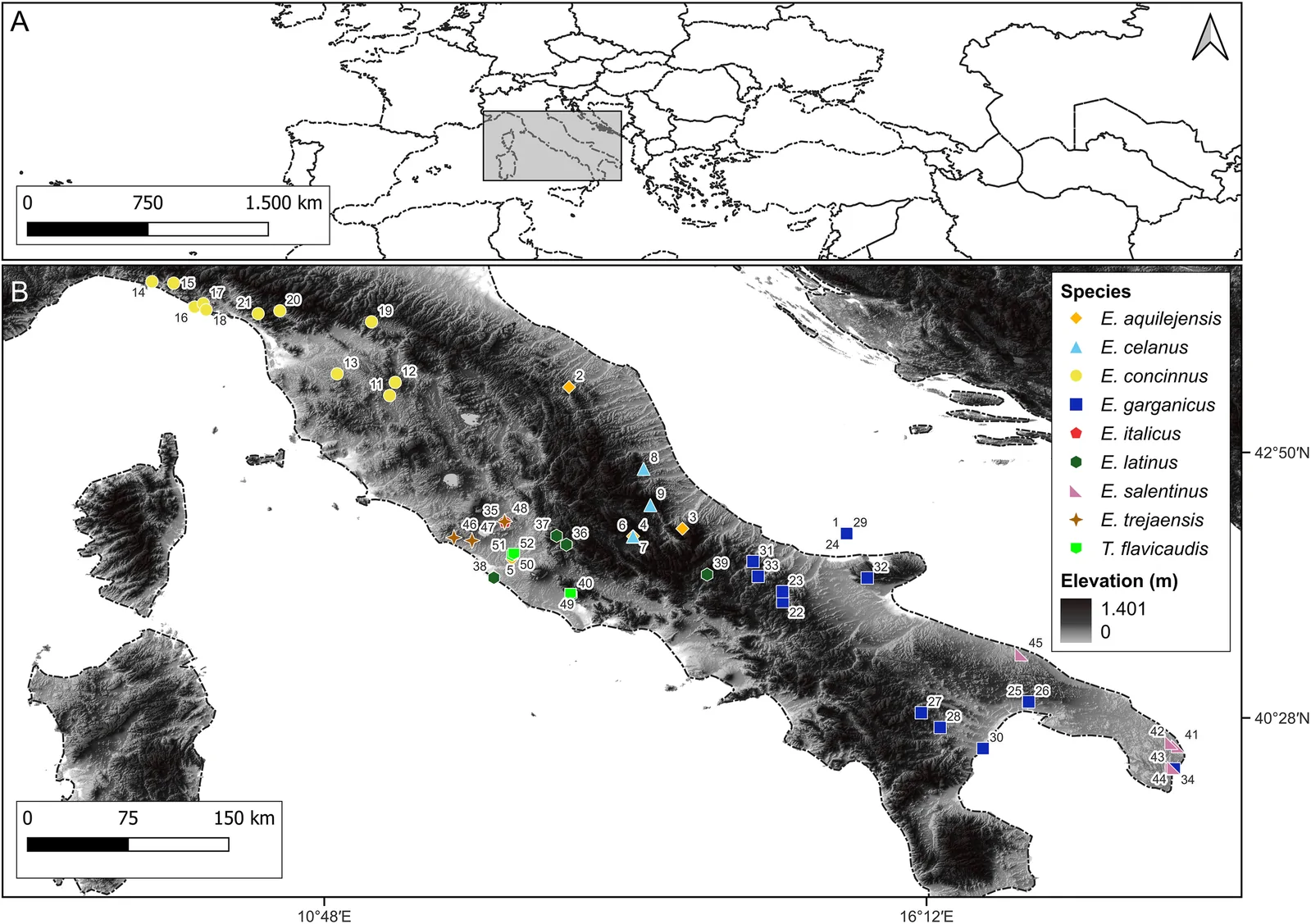
Map of the Italian Peninsula indicating sample localities, species of *Euscorpius*Thorell, 1876 and population codes (Table 1).

**Fig. 2:**
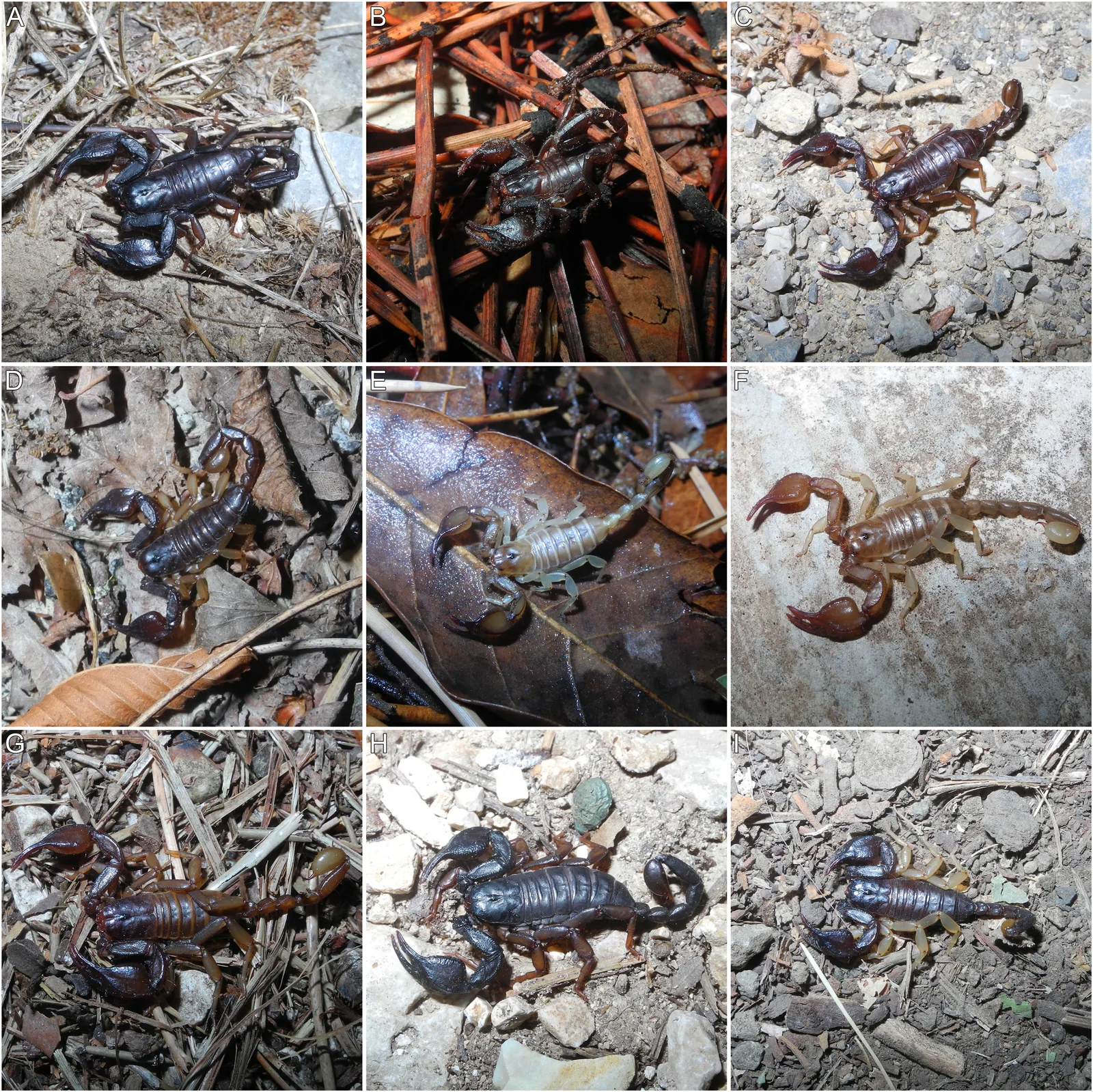
Adult males of nine Italian scorpion species sampled for study. **A.***Euscorpius concinnus* (Koch, 1837b). **B.***Euscorpius latinus*Tropea and Parmakelis, 2022. **C.***Euscorpius trejaensis*Tropea and Parmakelis, 2022. **D.***Euscorpius celanus*Tropea, 2012. **E.***Euscorpius garganicus*Caporiacco, 1950. **F.***Euscorpius salentinus* Tropea, 2017. **G.***Euscorpius aquilejensis* (Koch, 1837b). **H.***Euscorpius italicus* (Herbst, 1800). **I.***Tetratrichobothrius flavicaudis* (DeGeer, 1778).

Fifty-two localities (proxies for populations) were sampled, varying from two to thirteen per species (Fig. 1; Table 1). Species with broad geographical distributions, e.g., *E. garganicus*, were sampled across their entire range to maximize representation of venomic and genetic variation and are therefore represented by more collection localities (Fig. 1).

**Table 1:** Species and populations of *Euscorpius*Thorell, 1876 sampled across Italy, with codes for specimens used for DNA isolation (LP) and venom extraction (MR). Codes in corresponding positions refer to same individual (e.g., LP 18802 = MR 22, etc.). Underlined codes correspond to DNA sequences and venom mass fingerprints (MFPs) selected as reference profiles for each population. Mismatched underlined codes indicate DNA sequences and MFPs from different individuals within the same population.

Species	Population	DNA samples (LP)	Venom samples (MR)
*E. aquilejensis*	1	19018	299
	2	18896	146
	3	18905, 18906, 18907, 18908	157, 158, 159, 160
	4	18913	166
	5	19022, 19023, 19024	305, 306, 307
*E. celanus*	6	18801, 18802, 18803	21, 22, 23
	7	18804, 18805, 18806, 18807, 18808	25, 26, 27, 28, 29
	8	18874, 18875, 18876, 18877, 18878, 18879, 18880	119, 120, 121, 122, 123, 124, 125
	9	18898, 18899, 18900, 18901, 18902, 18903, 18904	149, 150, 151, 152, 153, 154, 155
	10	18909, 18910, 18911, 18912	162, 163, 164, 165
*E. concinnus*	11	18823, 18824, 18825, 188126, 18827	52, 53, 54, 55, 56
	12	18828, 18829, 18830, 18831, 18832	58, 59, 60, 61, 62
	13	18833, 18834, 18835, 18836, 18837	64, 65, 66, 67, 68
	14	18844, 18845, 18846, 18847, 18848	80, 81, 82, 83, 84
	15	18849, 18850, 18851, 18852, 18853	87, 88, 89, 90, 91
	16	18854, 18855, 18856, 18857, 18858	94, 95, 96, 97, 98
	17	18859, 18860, 18861, 18862	100, 101, 102, 103
	18	18863, 18864, 18865, 18866, 18867, 18868, 18869	106, 107, 108, 109, 110, 111, 112
	19	18870, 18871, 18872	114, 115, 116
	20	18838, 18839	70, 71
	21	18840, 18841, 18842, 18843	73, 75, 76, 77
*E. garganicus*	22	18923, 18924, 18925, 18926	179, 180, 181, 182
	23	18927, 18928	185, 186
	24	19014, 19015, 19016, 19017	295, 296, 297, 298
	25	18945, 18946, 18947, 18948, 18949	207, 208, 209, 210, 211
	26	18950, 18951, 18952, 18953, 18954	213, 214, 215, 216, 217
	27	18955, 18956, 18957, 18958, 18959, 18960, 18961	219, 220, 221, 222, 223, 224, 225
	28	18962, 18963, 18964	227, 228, 229
	29	19010, 19011, 19012, 19013	289, 290, 291, 292
	30	18965, 18966, 18967, 18968, 18969	232, 233, 234, 235, 236
	31	18919, 18920, 18921, 18922	174, 175, 176, 177
	32	18940, 18941, 18942, 18943, 18944	201, 202, 203, 204, 205
	33	18998, 18999, 19000	273, 274, 275
	34	18982, 18983, 18984, 18985	252, 253, 254, 255
*E. italicus*	35	18793, 18794, 18795, 18796, 18797, 18798, 18799	12, 13, 14, 15, 16, 17, 18
*E. latinus*	36	18809, 18810, 18811	32, 33, 34
	37	18812, 18813, 18814	36, 37, 38
	38	18881, 18882, 18883, 18884, 18885	127, 128, 129, 130, 131
	39	18914, 18915, 18916, 18917, 18918	168, 169, 170, 171, 172
	40	18815, 18816, 18817, 18818, 18819	41, 42, 43, 44, 45
*E. salentinus*	41	18970, 18971, 18972, 18973, 18974, 18975	238, 239, 240, 241, 242, 243
	42	18976, 18977, 18978, 18979, 18980, 18981	245, 246, 247, 248, 249, 250
	43	18986, 18987, 18988, 18989, 18990, 18991	257, 258, 259, 260, 261, 262
	44	18992, 18993, 18994, 18995	264, 265, 266, 267
	45	18996, 18997	269, 270
*E. trejaensis*	46	18886, 18887, 18888, 18889	133, 134, 135, 136
	47	18890	138
	48	18800	20
*T. flavicaudis*	49	18820, 18821, 18822	48, 49, 50
	50	18788, 18789, 18790, 18791, 18792	4, 5, 6, 7, 8
	51	19020, 19021	303, 304
	52	19019	301

### 2.2 Sex determination, specimen selection and venom extraction

Up to seven specimens per population were sampled for venom and subsequently preserved for DNA isolation (Table 1). Sexual and ontogenetic variation in venom composition has been documented in *Euscorpius*, with adult males exhibiting different profiles from adult females and immatures of both sexes (Krämer et al., 2022). Therefore, to avoid potential bias, only adult females and immatures, which share the same profile, were used in the study. Sex and life stage were determined based on the morphology of the genital operculum (presence or absence of genital papillae in males and females, respectively) and the presence or absence of secondary sexual characters of the pedipalp chela (lobes and notches on the fingers) and telson (swollen or not) that develop exclusively in adult males (Krämer et al., 2022; Blasco-Aróstegui and Prendini, 2023, 2024).

Venom samples were extracted in the field using electrical stimulation (Schaffrath and Predel, 2014; Carcamo-Noriega et al., 2019; Krämer et al., 2022). A custom-built “milking” apparatus was employed, comprising a Promed Tens device (Promed GmbH, Farchant, Germany) connected to metallic forceps, one to hold the scorpion at the base of the metasoma, and the other to apply direct current to the membrane between the telson and fifth metasomal segment (Schaffrath and Predel, 2014; Krämer et al., 2022). To minimize harm to specimens while maximizing venom yield, forceps were immersed in saline solution to improve electrical conductivity, and the following stimulation parameters applied: 16 V, 45 Hz, diphase fixe current set at 22 s, and 3 s pulses. Expelled venom was collected in glass capillary tubes (inner diameter 1 mm; Hilgenberg GmbH, Malsfeld, Germany) and transferred to 1.5 mL microtubes (Eppendorf, Hamburg, Germany) containing 200 μL of 35% EtOH.

An initial, preliminary venom extraction from each specimen was discarded, as it may differ in composition and volume among specimens due to prior feeding or defense (Carcamo-Noriega et al., 2019). After 24 h, all individuals were “milked” again and these samples retained for subsequent analysis (Schaffrath and Predel, 2014; Schaffrath et al., 2018; Krämer et al., 2022). Although not all venom components are fully regenerated within 24 h, species-specific signals appear to be mostly restored in *Euscorpius* (Krämer et al., 2022). The extent of variation in freshly regenerated venom collected 24 h after the initial stimulation vs after three, five and seven days has been evaluated in *E. italicus* (Krämer et al., 2022) and *T. flavicaudis* (Fig. S1) under controlled conditions.

Before initiating the final analyses, a series of dilutions was prepared for each sample: (1) STOCK dilution, comprising 9 μl of the original venom sample and 1 μl of 1% TFA; (2) 1:2 dilution, consisting of 1 μl of the STOCK dilution and 2 μl of DHB/EtOH mixture; (3) 1:5 dilution, consisting of 1 μl of the STOCK dilution and 4 μl of DHB/EtOH mixture; and (4) 1:10 dilution, consisting of 1 μl of the STOCK dilution and 9 μl of DHB/EtOH mixture. Based on preliminary tests, the 1:2 dilution was selected as optimal, and all subsequent analyses performed with it. All venom dilutions were stored at −20 °C.

### 2.3 MALDI-TOF MS

Approximately 0.4 μl of 10 mg/ml 2.5-dihydroxybenzoic acid (Sigma-Aldrich, Steinheim, Germany), dissolved in 20% acetonitrile/1% formic acid, was used as a matrix and mixed with the same quantity of each venom dilution directly on the sample plate for MALDI-TOF MS. The mixed solution was then air-dried. MALDI-TOF MS was conducted in reflectron positive ion mode on an UltrafleXtreme TOF/TOF mass spectrometer (Bruker Daltonik, Bremen, Germany) at the University of Cologne (UC), Germany. Samples were recorded in the mass ranges of *m/z* 800‒4500 and *m/z* 3000‒10,000. Peak matching and alignment were performed using the default mass tolerance of 50 ppm in ClinProTools (Bruker Daltonics, Billerica, MA). The number and intensity of laser shots were adjusted to obtain an optimal signal-to-noise ratio, with most data extracted from 10,000 shots and 666 Hz laser frequency. For external calibration, a mixture containing proctolin ([M+H]^+^, 649.3), Drm-sNPF-21^2−19^, ([M+H]^+^, 974.5), Pea-FMRFa-12 ([M+H]^+^, 1009.5), Lom-PVK ([M+H]^+^, 1104.6), Mas-allatotropin ([M+H]^+^, 1486.7), Drm- IPNa ([M+H]^+^, 1653.9), Pea-SKN ([M+H]^+^, 2010.9), and glucagon ([M+H]+, 3481.6) was used for the mass range of *m/z* 800–4500 and a mixture of bovine insulin ([M+H]^+^, 5731.5), glucagon and ubiquitin ([M+H]^+^, 8560.6) for the mass range of *m/z* 3000–10,000. Ion signals from the published MFP of *E. italicus*, which could be partially linked to the corresponding sequences (Krämer et al., 2022), were used for internal calibration.

### 2.4 MS raw data processing and filtering

Ion signals were identified using the peak detection algorithm CENTROID in flexAnalysis 3.4 (Bruker Daltonics). The CENTROID algorithm (with default parameters) was preferred, as it identifies and assigns masses to all peaks in the spectra, ensuring a more comprehensive and consistent detection of venom components. Nevertheless, as automated peak detection algorithms are not without limitations, all peaks detected were subsequently verified through manual inspection of spectra to ensure accurate peak assignment. Raw spectra were deposited at the ProteomeXchange Consortium via the Proteomics Identification Database (PRIDE) of the European Bioinformatics Institute (EMBL-EBI, accession code PXD084418; https://www.ebi.ac.uk/pride/).

Between 20 and 31 of the highest-intensity and most consistently present ion signals were selected among venom samples of the individuals representing each population (*n* = 1–7 individuals per population; Table 1). The resulting lists of ion signals per population were then compared among populations, prioritizing ion signals consistently present. When two or more ion signals fell within a similar signal intensity and mass range, all were retained, which accounts for variation in the number of masses detected among species. Once finalized for all species, a reference MFP per population was selected and the monoisotopic mass ion signals and corresponding intensities compiled into a master mass list. All detected ion signals were assembled, and each venom MFP scored for presence (1) or absence (0), producing a binary matrix that minimized variation due to experimental factors or variable venom expression (Table S1).

As venom samples were analyzed without prior chromatographic fractionation, the inherent ion suppression associated with MALDI-TOF MS was assumed. However, because closely related species are expected to possess broadly similar venom peptide repertoires, ion suppression is also expected to affect comparable classes of peptide ions across samples, thereby minimizing its influence on interspecific comparisons.

### 2.5 Assessment of intra- and interspecific venom diversity

The matrix of venom signal presence/absence was used to assess whether significant differences exist among conspecific and heterospecific populations. Non-metric multidimensional scaling (NMDS) analyses were performed as this ordination method is well suited for binary presence/absence data (Minchin, 1987). Three commonly used similarity/dissimilarity indices—i.e., Bray-Curtis (Bray and Curtis, 1957), Dice (1945), and Jaccard (1912)—widely considered appropriate for binary datasets and extensively applied in venom mass fingerprinting studies (Minchin, 1987; Schaffrath, 2017; Schaffrath et al., 2018; Zancolli et al., 2019; Avella et al., 2022, 2023; Ajdi et al., 2024), were also applied.

Preliminary analyses were conducted to determine which available dissimilarity measure best fit the data, by performing NMDS on each index and comparing the resulting stress values. Concordance among the resulting distance matrices was further assessed using Mantel tests (Spearman's rank correlation; Mantel, 1967; Dietz, 1983) to evaluate pairwise similarity and Procrustes randomization tests (PROTEST; Jackson, 1995) to assess overall concordance of ordination structures (both with 999 permutations). Based on these results, the most suitable distance measure was selected for subsequent analyses.

Permutational Multivariate Analysis of Variance (PERMANOVA; Anderson, 2001) was then applied to the chosen distance matrix with 9999 permutations to test whether species differed in multivariate venom composition beyond random expectations. As PERMANOVA may be sensitive to differences in within-group dispersion, homogeneity of multivariate dispersions among species was evaluated using a Permutational Analysis of Multivariate Dispersions (PERMDISP; Anderson, 2006) with 9999 permutations. Additionally, an Analysis of Similarities (ANOSIM; Field et al., 1982; Clarke, 1993) with 9999 permutations was performed as a complementary, rank-based assessment of overall group separation. All analyses were conducted in R version 4.5.2 using the ‘vegan’ 2.6–10 (Oksanen et al., 2022) and ‘pairwiseAdonis’ 0.4 (Arbizu, 2020) packages.

### 2.6 Hierarchical Clustering Analysis

Hierarchical Clustering Analyses (HCA) using Ward's (1963) method were conducted to assess whether the differences detected by NMDS were consistent with patterns previously inferred from DNA phylogenies (including the phylogeny generated in the present study, see below). Hierarchical Clustering Analysis was initially applied to the NMDS coordinates to provide a visual representation of clustering patterns in reduced ordination space. Additional HCAs were performed using several distance metrics employed in previous studies of venom MFPs (Schaffrath, 2017; Schaffrath et al., 2018; Ajdi et al., 2024), including Bray-Curtis (Bray and Curtis, 1957), Dice (1945), Euclidean, Gower-Legendre (Gower, 1971; Gower and Legendre, 1986), Intersection (derived from the intersection-over-union family associated with Jaccard/Soergel measures), Jaccard (1912), Kulczyński (1928), Manhattan (city-block *L*_1_), Rogers-Tanimoto (Rogers and Tanimoto, 1960), Simple Matching (Sokal and Michener, 1958) and Sokal-Sneath (Sokal and Sneath, 1963) distances. Preliminary analyses were used to determine the most suitable distance metric, based on cophenetic correlation values. Resulting dendrograms were compared with the phylogenetic tree topology to assess correspondence with venom composition patterns and guide the selection of a method that best preserved the distance structure (i.e., highest cophenetic correlation) and most closely reflected phylogenetic relationships (i.e., most biologically accurate).

### 2.7 DNA extraction, PCR amplification and sequencing

Genomic DNA was extracted from muscle tissue in the fourth leg of specimens from the same collection localities (i.e., same populations) as those analyzed for venom composition (Table 1). Whenever preservation conditions permitted, DNA was extracted from the same individuals from which representative venom MFPs were selected. DNA extractions followed the spin-column protocol of the Qiagen DNeasy Blood and Tissue Kit (Qiagen, Valencia, CA). Samples were digested with Proteinase K for 24–72 h in an incubator prior to DNA purification, and DNA subsequently eluted in 200 μL of AE buffer.

The mitochondrial COI gene was used as a DNA barcode for comparison with venom MFPs (i.e., venom barcodes), as it has been widely applied for species delimitation and phylogenetic inference in *Euscorpius* (Graham et al., 2012; Parmakelis et al., 2013; Fet et al., 2016; Podnar et al., 2021). PCR amplification of the COI gene was conducted in the Institute for Comparative Genomics at the American Museum of Natural History, New York, using previously established protocols (e.g., Prendini et al., 2003; Loria et al., 2022; Blasco-Aróstegui et al., 2025). The amplification was divided into two fragments, with reagents and protocols optimized for each primer pair, including EmeraldAmp® DNA Polymerase (Clontech, Takara Bio, New Delhi, India). Forward primers included: COImodF (5′-ATCATAAGGATATTGGGACTATGT-3'; Bryson et al., 2013), LCO (5′-GGTCAACAAATCATAAAGATATTGG-3'; Folmer et al., 1994), CI-J01718 (5′-GGNGGATTTGGAAATTGRTTRGTTCC-3'; Simon et al., 1991), and EXTA (5′-GAAGTTTATATTTTAATTTTACCTGG-3'; Prendini et al., 2003). Reverse primers included: HCOoutout (5′-GTAAATATATGRTGDGCTC-3'; Prendini et al., 2003), HCO (5′-TAAACTTCAGGGTGACCAAAAAATCA-3'; Folmer et al., 1994), CI-N02776 (5′-GGATAATCAGAATANCGNCGAGG-3'; Simon et al., 1991), and EXTB (5′-CCTATTGAWARAACATARTGAAAATG-3'; Prendini et al., 2003).

COI amplicons were verified under ultraviolet light on 4% agarose gels stained with SYBR Safe DNA (ThermoFisher Scientific, Waltham, MA). PCR products were purified using the AMPure XP system (Beckman Coulter, Brea, CA) and sequenced with the STeP50 Program (Platt et al., 2007) using Eppendorf EP and Pro thermocyclers (Hamburg, Germany). Sanger dideoxy sequencing was performed with a BigDye Terminator 3.1 Cycle Sequencing Kit (Life Technologies, Carlsbad, CA) on an ABI 3730XL automated DNA sequencer (PerkinElmer, Waltham, CA).

Sequences were edited and assembled using Sequencher 5.4.6 (Gene Codes Corporation, Ann Arbor, MI; http://www.genecodes.com) and deposited in GenBank (https://www.ncbi.nlm.nih.gov/genbank; Table S2). The dataset comprised 52 COI sequences (each 1078 base-pairs in length) representing populations sampled across the Italian Peninsula, together with one COI sequence from a Slovenian population of *E. italicus* (RP1), extracted from the transcriptome assembly of Krämer et al. (2022) (GenBank accession GKBM01020311.1) and included as a reference in both the molecular and venom datasets.

### 2.8 Phylogenetic analysis

Phylogenetic reconstruction was performed separately on the matrix of venom MFPs and the COI dataset using Maximum Likelihood (ML) in IQ-TREE 2.2.2.6 (Minh et al., 2020) (Table 1). For the DNA dataset, the best-fitting nucleotide substitution model was selected using ModelFinder (Kalyaanamoorthy et al., 2017) with the Bayesian Information Criterion (BIC). For the binary matrix of venom MFPs, ModelFinder was applied with the -st BIN flag to test all available binary models under the BIC. In this context, ‘0’ represents the ancestral condition and ‘1’ the derived condition, corresponding mainly to neomorphic (presence/absence) characters (Sereno, 2007) hence allowing for asymmetric state frequencies is appropriate (e.g., Pyron, 2017; Sun et al., 2018). Models TIM3+F + G4 and GTR2+FO (Kalyaanamoorthy et al., 2017; Minh et al., 2020) were selected for the DNA and venom datasets, respectively.

Branch support was assessed using 10,000 replicates of the ultrafast bootstrap approximation (UFBoot; Minh et al., 2013; Hoang et al., 2018), with subsequent refinement using nearest-neighbor interchange (NNI) to reduce potential overestimation of support (Sankoff et al., 1994; Minh et al., 2020). An SH-aLRT test (Guindon et al., 2010) with 10,000 replicates was also conducted to provide complementary support values.

The resulting tree files were visualized and edited in FigTree 1.4.4, with branches shown in ‘proportional’ mode to reflect the most similar structure to the resulting dendrograms of hierarchical cluster analyses of venom MFPs. Intra- and interspecific genetic distances were estimated in MEGA12 (Kumar et al., 2024).

## 3 Results

### 3.1 Analysis of mass spectra

A total of 210 venom samples were analyzed (Table 1) and the corresponding mass spectra visualized and compared, initially among populations and subsequently among species. After data filtering, a single representative MFP was selected for each species and population. These profiles were then used to score each population in the binary matrix. Selected examples of species-specific venom MFPs are illustrated in Fig. 3, Fig. 4, Fig. 5, Fig. 6, Fig. 7, Fig. 8.

**Fig. 3:**
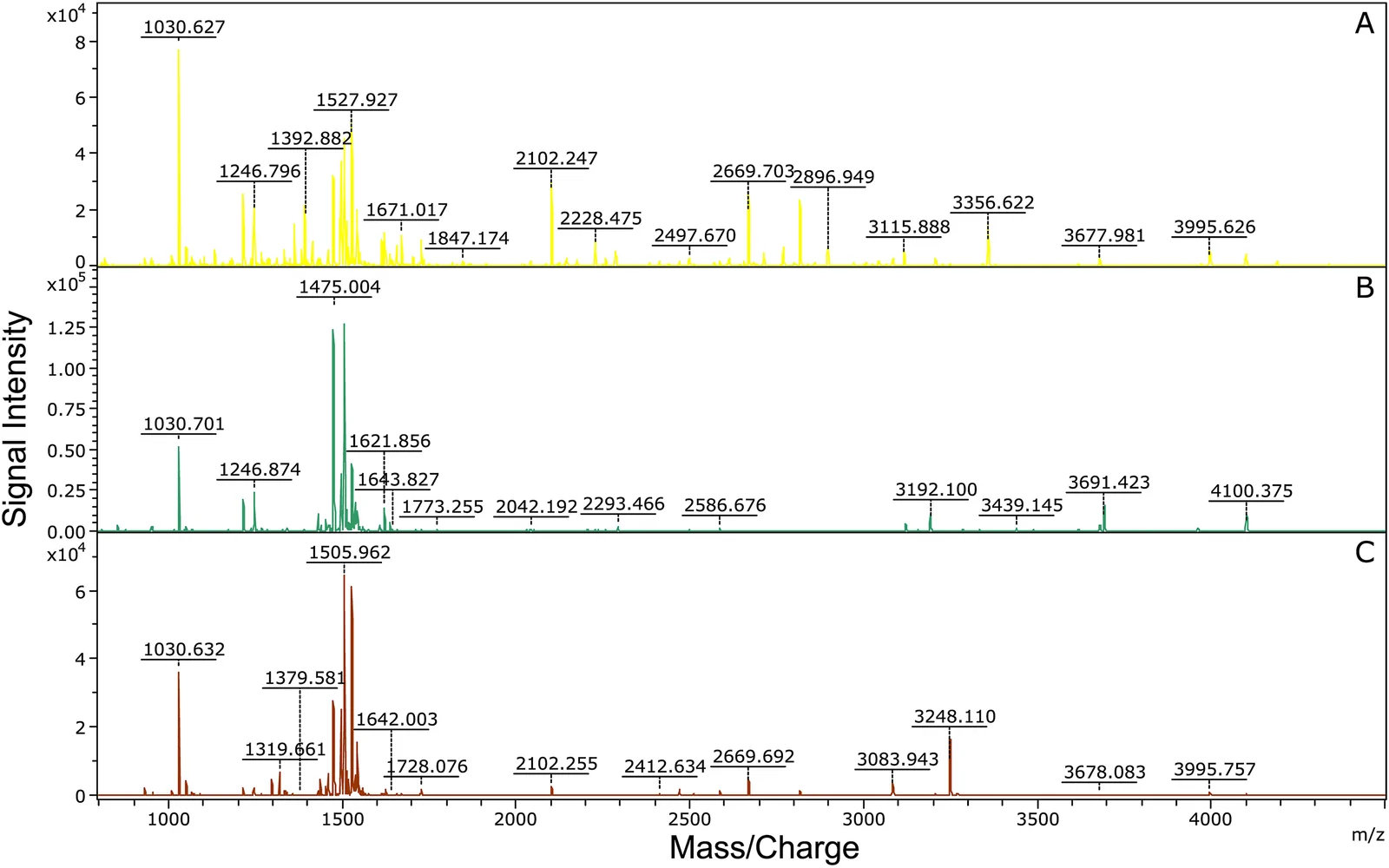
MALDI-TOF venom mass fingerprints (MFPs) obtained from diluted venom samples of individuals representing three Italian species of *Euscorpius concinnus* (Koch, 1837b) complex (*m/z* 800‒4500; prominent ion signals labeled). **A.***Euscorpius concinnus*: MR54. **B.***Euscorpius latinus*Tropea and Parmakelis (2022): MR170. **C.***Euscorpius trejaensis*Tropea and Parmakelis (2022): MR138.

**Fig. 4:**
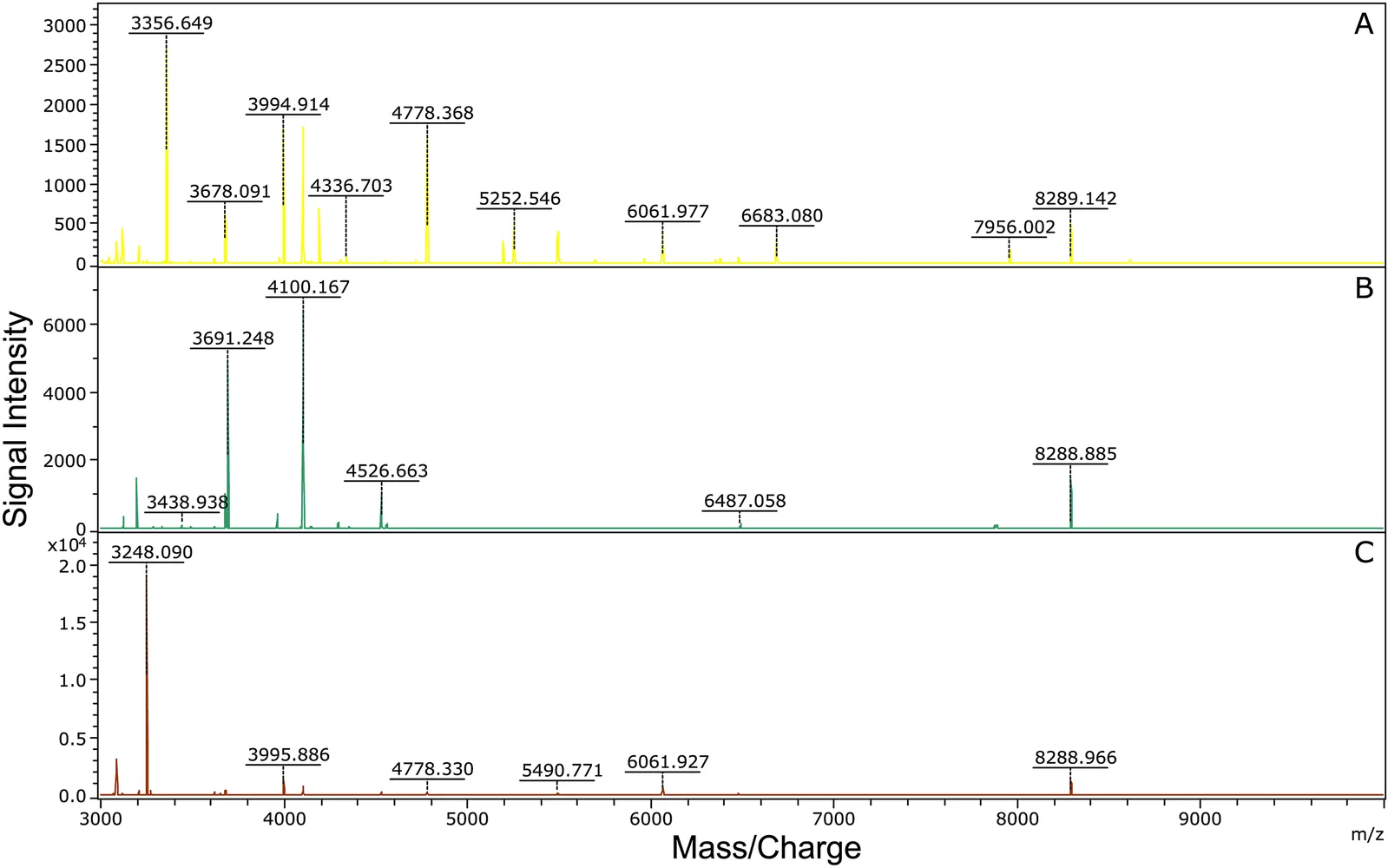
MALDI-TOF venom mass fingerprints (MFPs) obtained from diluted venom samples of individuals representing three Italian species of *Euscorpius concinnus* (Koch, 1837b) complex (*m/z* 3000‒10,000; prominent ion signals labeled). **A.***Euscorpius concinnus*: MR54. **B.***Euscorpius latinus*Tropea and Parmakelis, 2022: MR170. **C.***Euscorpius trejaensis* (Tropea and Parmakelis, 2022): MR138.

**Fig. 5:**
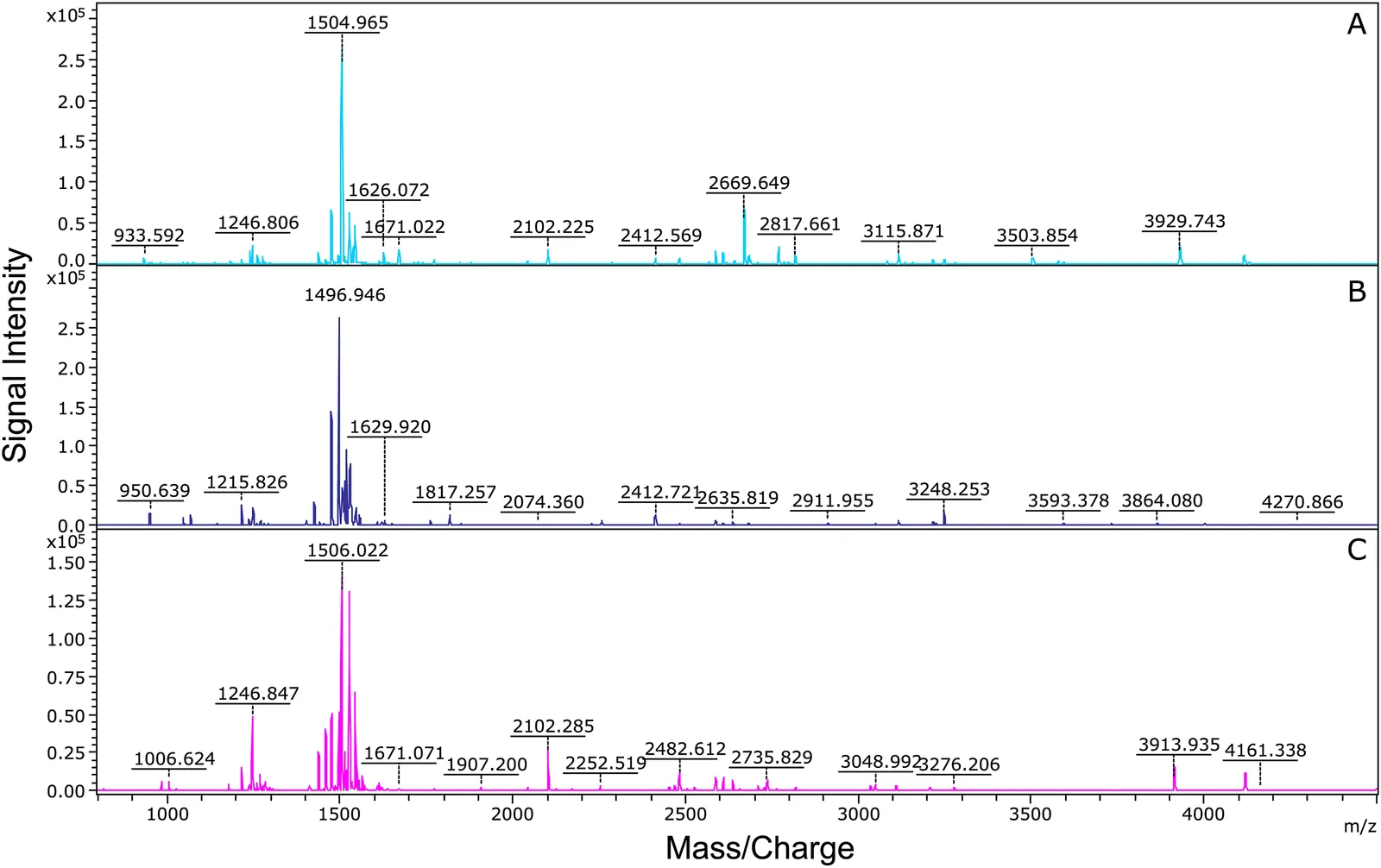
MALDI-TOF venom mass fingerprints (MFPs) obtained from diluted venom samples of individuals representing three Italian species of *Euscorpius sicanus* (Koch, 1837b) complex (*m/z* 800‒4500; prominent ion signals labeled). **A.***Euscorpius celanus*Tropea, 2012: MR163. **B.***Euscorpius garganicus*Caporiacco, 1950: MR295. **C.***Euscorpius salentinus* Tropea, 2017: MR269.

**Fig. 6:**
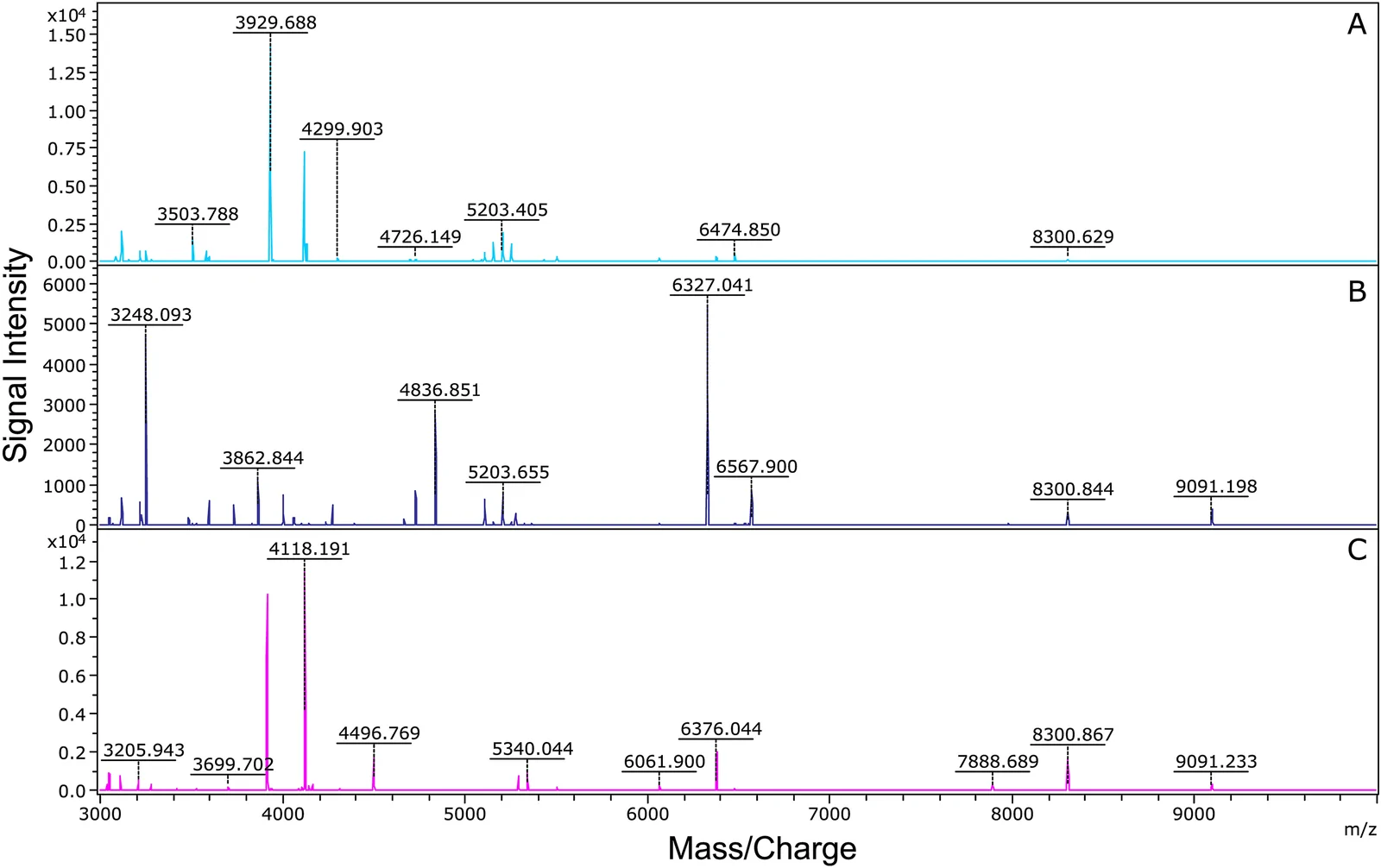
MALDI-TOF venom mass fingerprints (MFPs) obtained from diluted venom samples of individuals representing three Italian species of *Euscorpius sicanus* (Koch, 1837b) complex (*m/z* 3000‒10,000; prominent ion signals labeled). **A.***Euscorpius celanus*Tropea, 2012: MR163. **B.***Euscorpius garganicus*Caporiacco, 1950: MR295. **C.***Euscorpius salentinus*Tropea, 2017: MR269.

**Fig. 7:**
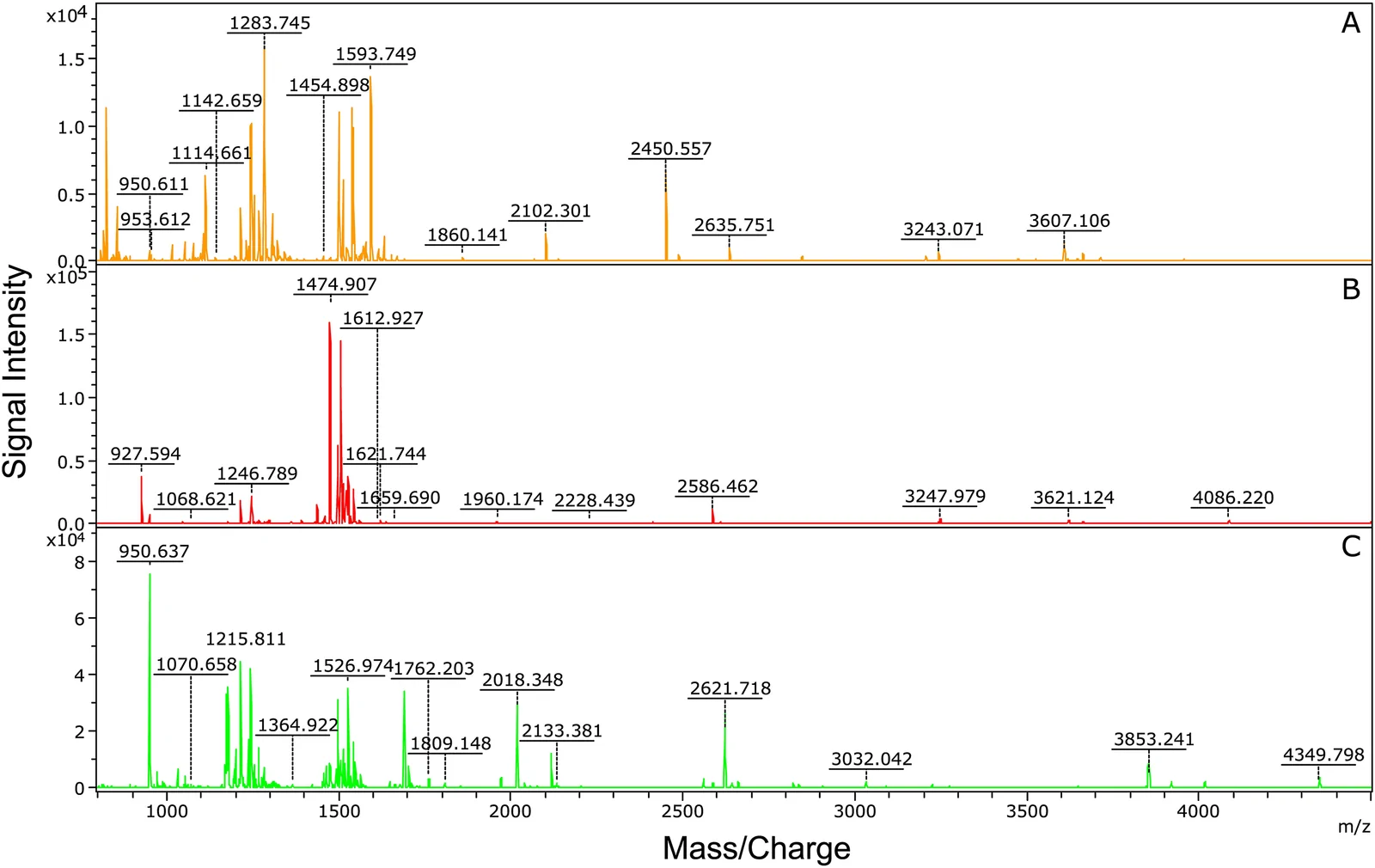
MALDI-TOF venom mass fingerprints (MFPs) obtained from diluted venom samples of two Italian species of *Euscorpius*Thorell, 1876 and *Tetratrichobothrius flavicaudis* (DeGeer, 1778) (*m/z* 800‒4500; prominent ion signals labeled). **A.***Euscorpius aquilejensis* (Koch, 1837b): MR305. **B.***Euscorpius italicus* (Herbst, 1800): MR16. **C.***Tetratrichobothrius flavicaudis*: MR303.

**Fig. 8:**
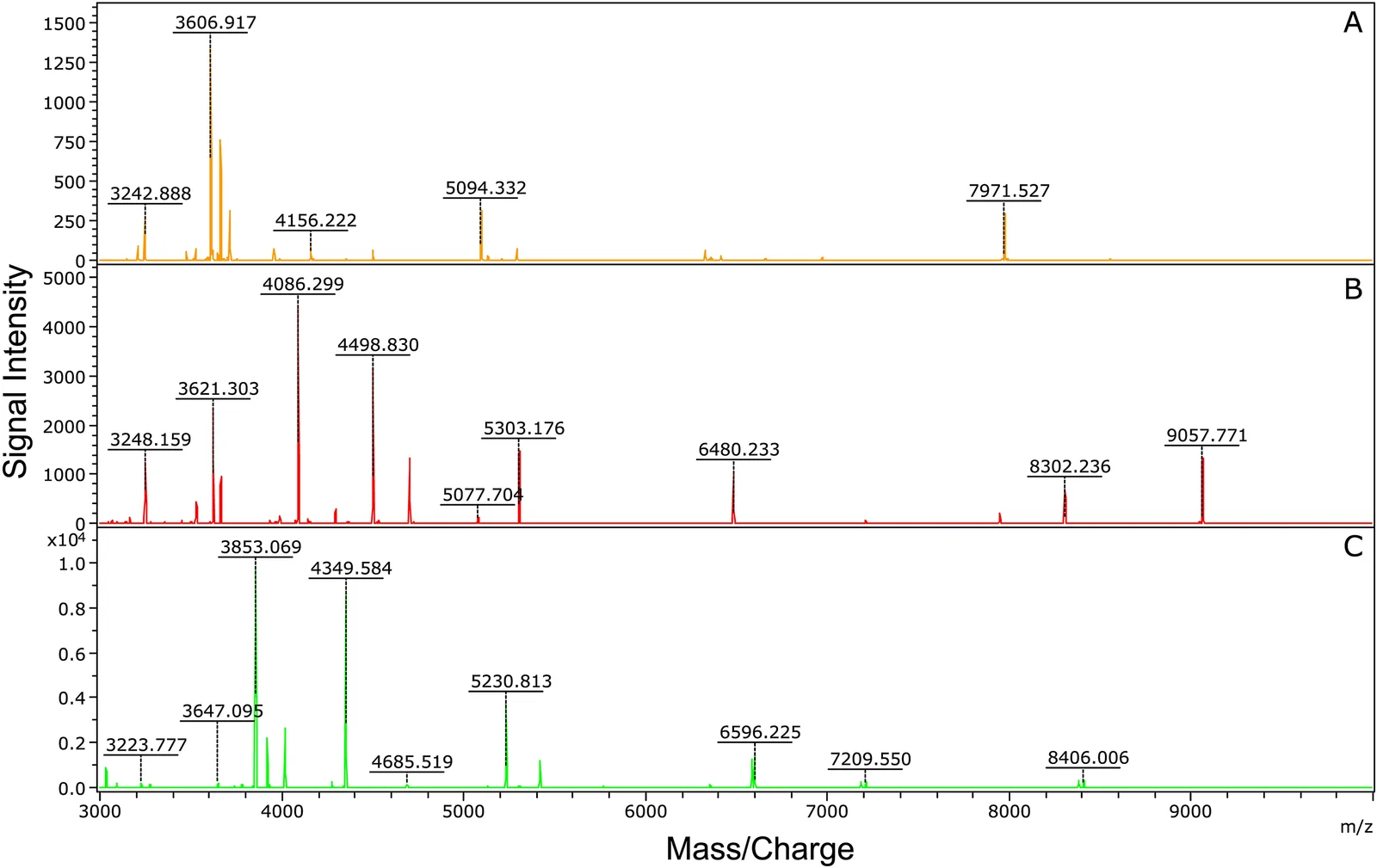
MALDI-TOF venom mass fingerprints (MFPs) obtained from diluted venom samples of two Italian species of *Euscorpius*Thorell, 1876 and *Tetratrichobothrius flavicaudis* (DeGeer, 1778) (*m/z* 3000‒10,000; prominent ion signals labeled). **A.***Euscorpius aquilejensis* (Koch, 1837b): MR305. **B.***Euscorpius italicus* (Herbst, 1800): MR16. **C.***Tetratrichobothrius flavicaudis*: MR303.

### 3.2 Assessment of intra- and interspecific venom diversity

The binary matrix used to assess the intra- and interspecific diversity in venom composition included 304 polymorphic ion signals distributed among the eight species of *Euscorpius* and outgroup, *T. flavicaudis*. The number of peaks selected per specimen ranged from six (e.g., *E. italicus* MR16S) to 31 (e.g., *E. garganicus* MR179), with an average of 12.3 peaks per individual across the 52 selected mass spectra analyzed (Table S1).

The NMDS stress values were identical for all three measures (Bray-Curtis, Dice and Jaccard; stress = 0.1219), indicating that each method adequately represented differences among venom profiles (Fig. S2). Mantel tests revealed strong correlations among all three distance matrices. Bray-Curtis, Jaccard and Dice were perfectly correlated (r = 1.0, p < 0.001), reflecting their monotonic mathematical relationship for binary data, as functionally related coefficients yield identical ordinations (Legendre and Legendre, 2012). PROTEST confirmed these patterns: all ordination configurations were identical (correlation = 1.0, p < 0.001). Jaccard's coefficient was ultimately selected as the primary measure for hypothesis testing, as it is more appropriate for strict binary presence/absence data and does not count shared absences as similarity, i.e., all terms have equal weight (Legendre and Legendre, 2012).

Seven main venom-based spatial clusters were retrieved in the NMDS ordination plot based on individual scores along the first two axes, i.e., NMDS1 and NMDS2, (Fig. 9). Whereas deeply divergent species, i.e., *E. aquilejensis*, *E. celanus*, *E. italicus*, and *T. flavicaudis*, formed distinct, well-separated clusters, species within the *concinnus* and *sicanus* complexes were grouped into two broader, partially overlapping clusters. Within the *concinnus* complex, the venom MFPs of *E. concinnus* formed a distinct cluster, whereas those of *E. latinus* and *E. trejaensis* partially overlapped. More overlap was observed among the MFPs of some related species of the *sicanus* complex, i.e., *E. garganicus* and *E. salentinus*. These patterns are consistent with the morphological and genetic affinities among the species of each complex.

**Fig. 9:**
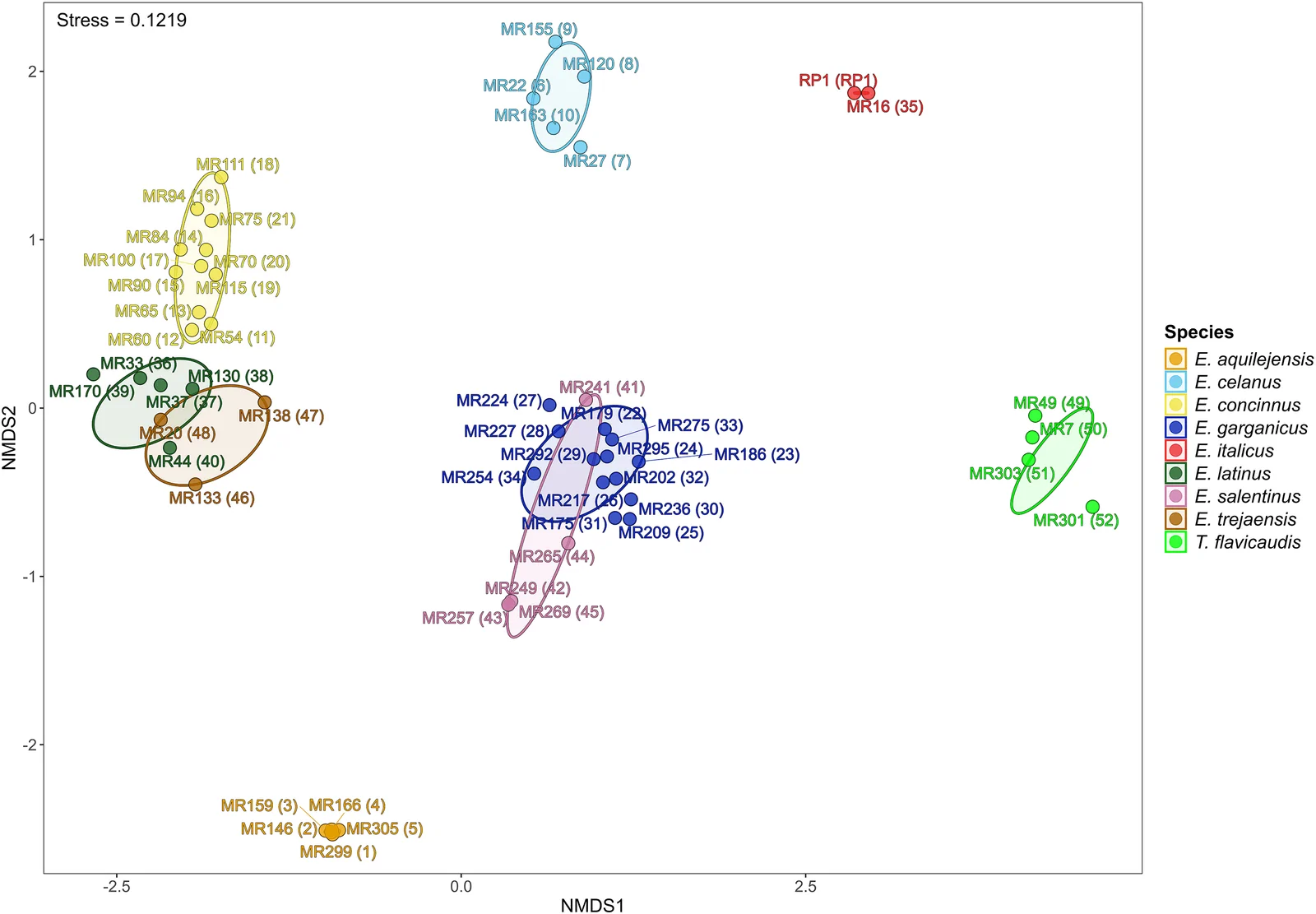
Non-metric multidimensional scaling ordination based on Jaccard distance (stress = 0.121) of 54 venom mass fingerprints (MFPs) from eight Italian species of *Euscorpius*Thorell, 1876 and *Tetratrichobothrius flavicaudis* (DeGeer, 1778). Each point represents individual from populations in Table 1. MR codes correspond to venom samples whereas numbers in parentheses indicate populations of origin. MFP of Slovenian population of *Euscorpius italicus* (Herbst, 1800) (RP1) from Krämer et al. (2022) included as reference.

The PERMANOVA performed on the Jaccard distance matrix confirmed that species clusters observed in the NMDS ordination were statistically significant (Adonis, *df* = 8, χ^2^ = 14.57, *F* = 10.46, *p* < 0.001). Species identity explained 65.5% of the total variance in venom composition (*R*^*2*^ = 0. 65543). Complementary ANOSIM results supported the marked separation among most species groups (R = 0.9773, *p* < 0.001), despite being more sensitive to heterogeneity in dispersions (Anderson and Walsh, 2013). The PERMDISP analyses revealed significant heterogeneity in multivariate dispersions among species (F = 7.51, p < 0.001), however, suggesting that some species exhibit greater intraspecific venom variation than others, e.g., *E. aquilejensis* vs. *E. garganicus* (Fig. S3; Table S3).

### 3.3 Hierarchical Clustering Analysis

All HCAs recovered two major clades, corresponding to the *concinnus* and *sicanus* species complexes (Fig. 10, Fig. 11). The HCA based on NMDS ordination space produced a clustering pattern largely congruent with the currently accepted phylogenetic relationships of *Euscorpius* (Tropea, 2017; Podnar et al., 2021; Tropea and Parmakelis, 2022), except for a few differences (Figs. 10 and 12‒14A). For example, the highly divergent *E. italicus* was placed within the *sicanus* clade, sister to *E. celanus*, which appears to represent the basal-most lineage of the *sicanus* complex in Italy (Fig. 10). Some *E. salentinus* samples clustered with one of the two subclades of *E. garganicus*. A similar pattern was observed for a single *E. latinus* sample, which grouped with *E. trejaensis*.

**Fig. 10:**
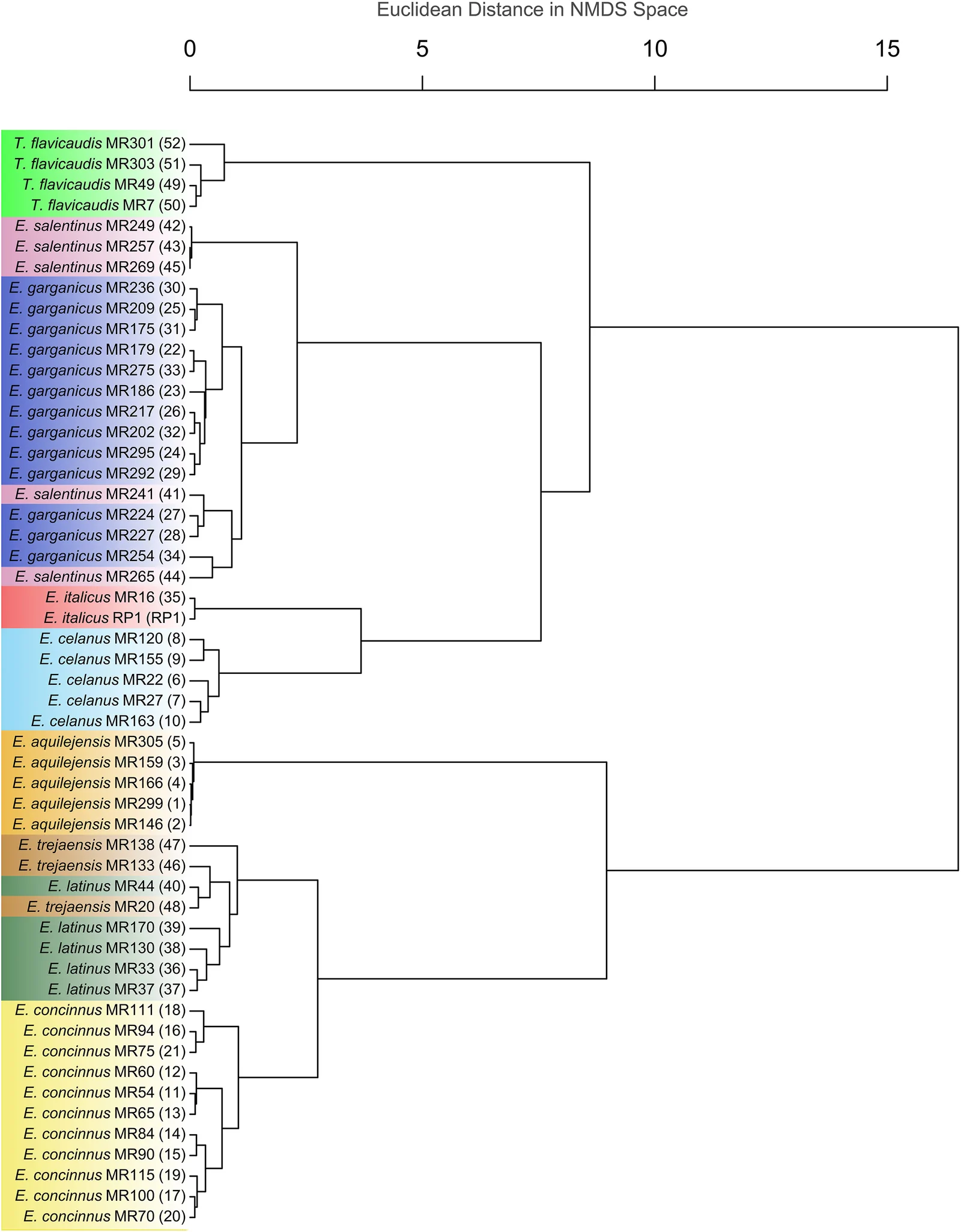
Hierarchichal clustering analysis using Ward's method based on Non-metric Multidimensional Scaling ordination space of 52 venom mass fingerprints (MFPs) from eight Italian species of *Euscorpius*Thorell, 1876 and *Tetratrichobothrius flavicaudis* (DeGeer, 1778). Each tip represents MFP of individual from populations in Table 1. MR codes correspond to venom samples whereas numbers in parentheses indicate populations of origin. MFP of Slovenian population of *Euscorpius italicus* (Herbst, 1800) (RP1) from Krämer et al. (2022) included as reference.

**Fig. 11:**
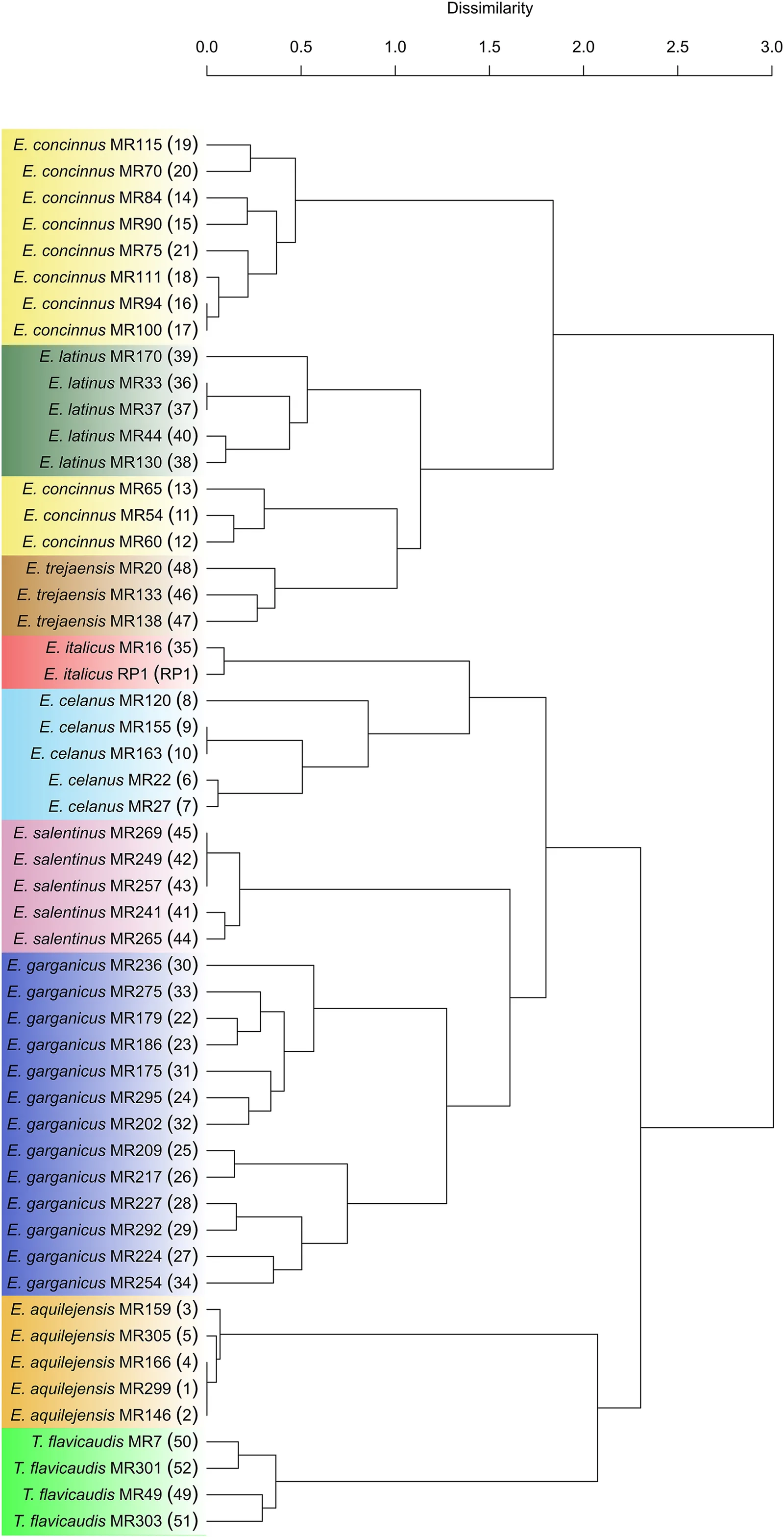
Hierarchichal clustering analysis using Ward's method and Intersection-based distance measure of 52 venom mass fingerprints (MFPs) from eight Italian species of *Euscorpius*Thorell, 1876 and *Tetratrichobothrius flavicaudis* (DeGeer, 1778). Each tip represents MFP of individual from populations in Table 1. MR codes correspond to venom samples whereas numbers in parentheses indicate populations of origin. MFP of Slovenian population of *Euscorpius italicus* (Herbst, 1800) (RP1) from Krämer et al. (2022) included as reference.

**Fig. 12:**
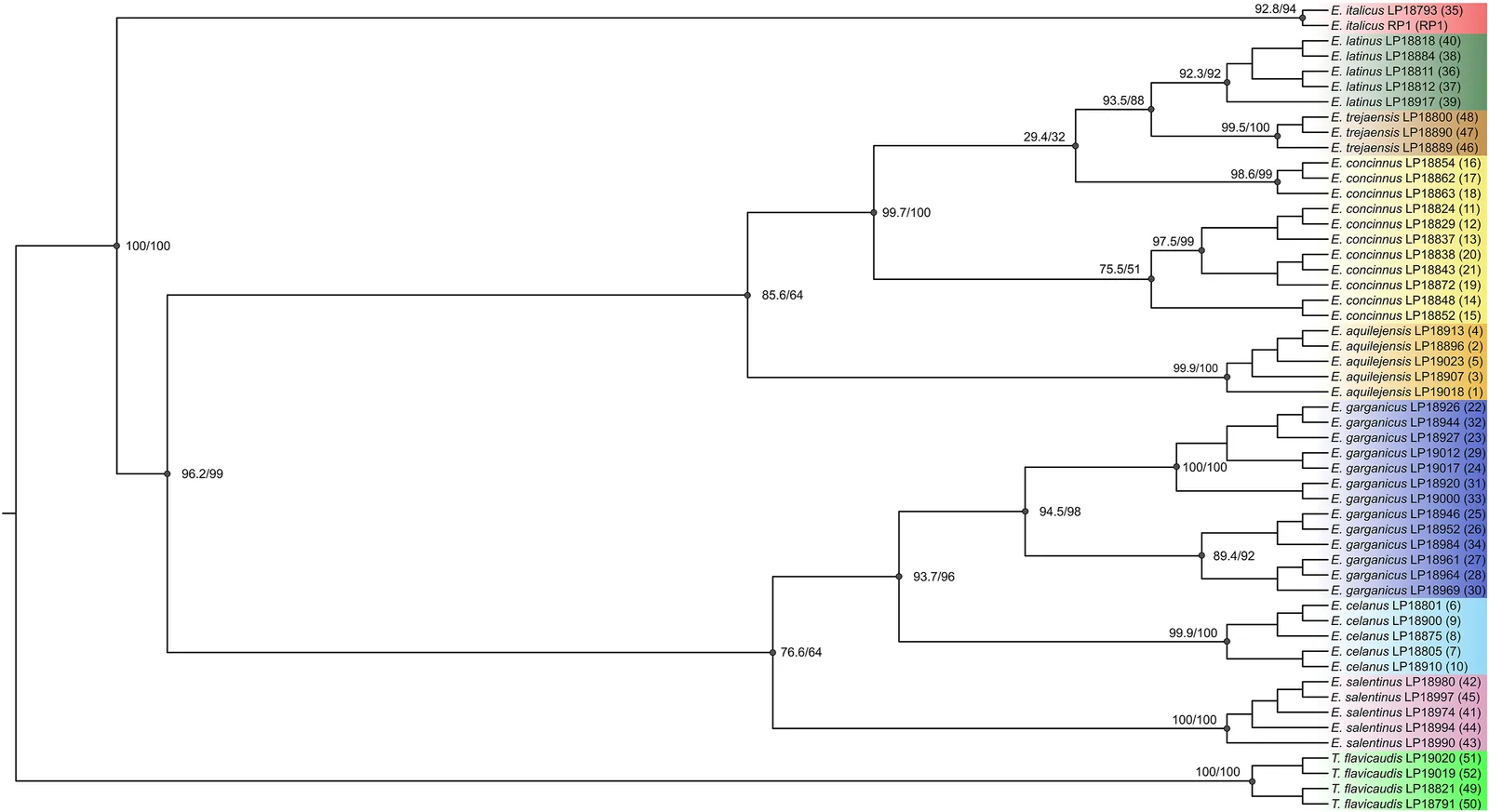
Maximum Likelihood phylogeny of eight Italian species of *Euscorpius*Thorell, 1876 and *Tetratrichobothrius flavicaudis* (DeGeer, 1778), inferred from DNA sequences of Cytochrome *c* Oxidase Subunit I (COI). Clade colors correspond to collection localities in Fig. 1. Specimen identifiers correspond to Table 1, except for the COI sequence of Slovenian *Euscorpius italicus* (Herbst, 1800) (RP1) from Krämer et al. (2022), included as a reference. Numbers on branches indicate bootstrap support values. (For interpretation of the references to color in this figure legend, the reader is referred to the online version of this article.)

**Fig. 13:**
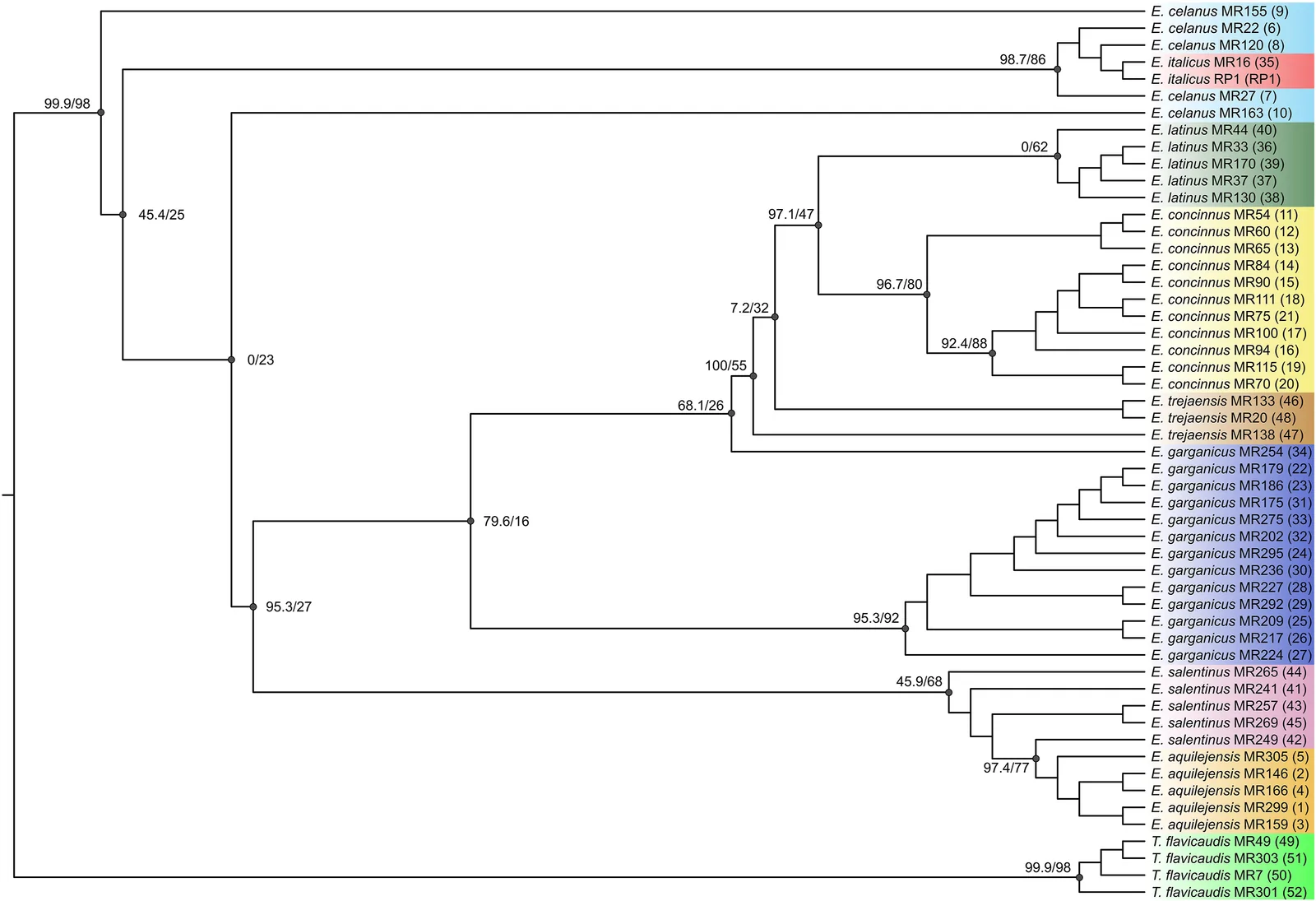
Maximum Likelihood phylogeny of eight Italian species of *Euscorpius*Thorell, 1876 and *Tetratrichobothrius flavicaudis* (DeGeer, 1778), inferred from 52 venom mass fingerprints (MFPs). Each tip represents MFP of individual from populations in Table 1. MR codes correspond to venom samples whereas numbers in parentheses indicate populations of origin. MFP of Slovenian population of *Euscorpius italicus* (Herbst, 1800) (RP1) from Krämer et al. (2022) included as reference.

**Fig. 14:**
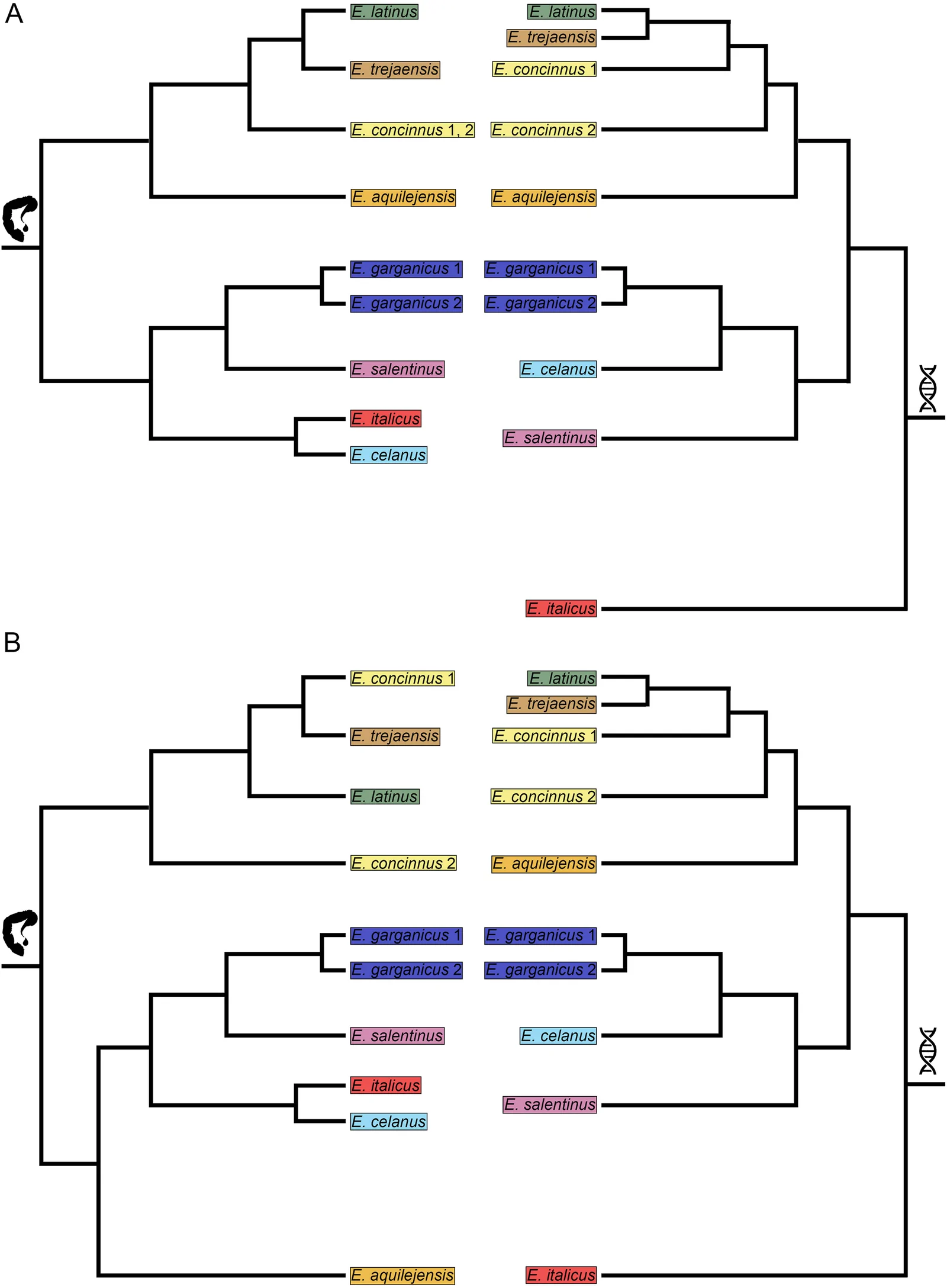
Simplified comparison between venom and DNA barcode tree topologies obtained by phylogenetic analysis of eight Italian species of *Euscorpius*Thorell, 1876, in each case with single representatives of major species clade indicated, species recovered in independent subclades represented separately (i.e., *E. concinnus* 1, *E. concinnus* 2, *E. garganicus* 1, and *E. garganicus* 2), and *Tetratrichobothrius flavicaudis* (DeGeer, 1778) pruned. **A.** Hierarchical clustering analysis (HCA) using Ward's method based on Non-metric Multidimensional Scaling ordination space (left) vs Maximum Likelihood (ML) phylogeny based on Cytochrome *c* Oxidase Subunit I (COI, right). **B.** HCA analysis using Ward's method and Intersection-based distance measure (left) vs ML phylogeny based on COI (right).

The HCA based on independent distance measures consistently recovered species-level clusters corresponding to putative clades, except for *E. concinnus*, which was subdivided into two distinct subclades (Fig. 11). Cophenetic correlation coefficients were consistently high across methods (>0.8) with lowest fit for the Sokal-Sneath distance (0.83; Fig. S4). The highest cophenetic correlations, obtained with the Gower-Legendre, Manhattan and Simple Matching coefficients (0.85; Fig. S4), indicated a better fit to the original distance matrices. However, as the differences among methods were minimal, the Intersection-based distance measure (cophenetic correlation = 0.84) was ultimately selected, as it was consistently congruent with established phylogenetic hypotheses of *Euscorpius* (Fig. 11‒14B), differing primarily in the erroneous placements of *E. celanus* and *E. italicus* in the *sicanus* clade, and of *E. aquilejensis* sister to the outgroup, *T. flavicaudis*.

### 3.4 Phylogenetic analysis

Phylogenetic reconstruction using ML on the DNA dataset recovered three main clades: the *concinnus* complex, comprising *E. concinnus*, *E. latinus*, and *E. trejaensis*, with *E. aquilejensis* recovered as the basal species; the *sicanus* complex, comprising *E. celanus*, *E. garganicus*, and *E. salentinus*; and *E. italicus*, placed basal-most in the phylogeny (Fig. 12). These results are congruent with previous studies based on morphology and DNA sequence data (Tropea, 2017; Podnar et al., 2021; Tropea and Parmakelis, 2022). Phylogenetic reconstruction of the venom MFPs with ML recovered four main clades, along with several single-branch clades represented by individuals of *E. celanus* (Fig. 13). Only the *concinnus* complex was congruent with the DNA-based topology, however.

*Euscorpis concinnus* and *E*. *garganicus* were each subdivided into two distinct subclades in the DNA-based phylogeny, consistent with the patterns recovered with HCA based on venom MFPs, suggesting genetic structure likely associated with geographical differentiation (Fig. 1, Fig. 12). *Euscorpius concinnus* was also subdivided into two subclades in the venom-based phylogeny, but these subclades were less differentiated than those recovered by HCA (Fig. 11) and the DNA-based phylogeny (Fig. 12), and *E. garganicus* was unresolved (Fig. 13). These results, together with the placement of *E. celanus* with *E. italicus* and an *E. garganicus* specimen within the *concinnus* complex, indicate lower phylogenetic signal in venom-based dataset.

Bootstrap support values for nodes in the DNA-based phylogeny were generally high (Fig. 12), except for the clade comprising *E. latinus* and *E. trejaensis*, the first subclade of *E. concinnus* (29.4/32), and a minor subdivision of the latter subclade (75.5/51). In contrast, bootstrap support values for the venom-based phylogeny were generally low for deeper relationships and comparatively higher within subclades. Overall, mixed-species clustering was associated with lower node support across the topology (Fig. 13). Intra- and interspecific COI genetic distances are presented in Table S4.

## 4 Discussion

### 4.1 Intraspecific variation among Italian Euscorpius

Intraspecific variation in venom is widespread across the animal kingdom, reflecting the intrinsic plasticity of venom systems and the influence of multiple, often interacting, selection pressures such as diet, ecology, geography, and genetics (Morgenstern and King, 2013; Mille et al., 2014; Gangur et al., 2017; Balchan et al., 2026). In scorpions, this variation has been associated with biotope differences in *Scorpio palmatus* (Ehrenberg, 1828; (Abdel-Rahman et al., 2009), experimentally-induced dietary shifts in *Androctonus finitimus* (Pocock, 1897), *Hottentotta tamulus* (Fabricius, 1798) and *Tityus serrulatus* Lutz and Mello, 1922 (Pucca et al., 2014; Tobassum et al., 2018), or geographical structuring across populations of the genera *Opistophthalmus* Koch, 1837c, *Parabuthus* Pocock, 1890, and *Uroplectes* Peters, 1861 (Schaffrath et al., 2018). The role of phylogeny in shaping venom diversity remains underexplored by comparison (Zhijian et al., 2006).

In two of the species examined, genetic structure appeared to play a dominant role in shaping venom variation. *Euscorpius aquilejensis* and *E. italicus*, both characterized by low genetic divergence across their ranges (Fet et al., 2006, Fig. 12; Table S4), exhibited highly conserved venom profiles with negligible intraspecific variation (Fig. 9, Fig. 10, Fig. 11, S3; Table S3). The apparent ecological generalism, widespread distributions, and synanthropic tendencies of these species may be responsible for the low venom diversity observed. Both species are known to occupy a range of habitats, including natural forests and human-modified environments (e.g., crevices in abandoned buildings), which may facilitate dispersal and gene flow (Crucitti et al., 1998; Fet et al., 2006; Tropea, 2013). Selection may favor generalized venom phenotypes in these opportunistic species with catholic diets, as in several snake taxa (Margres et al., 2015, 2019; Hofmann et al., 2018; Rautsaw et al., 2019).

In contrast, taxa within the *concinnus* and *sicanus* complexes exhibit pronounced intraspecific heterogeneity and shared venom components (Fig. 9, Fig. 10, Fig. 11, S3; Table S3). This variation closely reflects their geographical distributions and is partly concordant with the genetic structure recovered by the COI phylogeny, suggesting a strong evolutionary signal in venom differentiation (Fig. 12; Table S4). Comparable geographical patterns in venom composition have been reported in African scorpions (e.g., Schaffrath et al., 2018; Ajdi et al., 2024), although without a DNA-based comparison. *Euscorpius concinnus* displayed extensive dispersion in NMDS space, supported by the PERMDISP results, consistent with intraspecific variation (Fig. 9 and S3; Table S3). The HCA also revealed notable dissimilarity among conspecific samples (Fig. 11, Fig. 14). Similar, albeit less pronounced, patterns were observed in *E. latinus* and *E. trejaensis*. A stronger signal emerged in the *sicanus* complex, where *E*. *garganicus* displayed marked intraspecific venom variation in the NMDS plot (Fig. 9) and the PERMDISP analysis (Fig. S3; Table S3), consistent with pronounced phylogenetic structure (Fig. 12; Table S4). Despite less phylogenetic structure, *E. celanus* also exhibited considerable venom variation, as indicated by the NMDS, PERMDISP values, and high intraspecific dissimilarity in the HCA (Fig. 9, Fig. 10, Fig. 11, S3; Table S3). Intraspecific variation was more moderate in *E. salentinus* by comparison.

An intermediate pattern, lacking either the conservatism of *E. aquilejensis* and *E. italicus* or the structure of *E. garganicus*, was observed in the outgroup, *T. flavicaudis*. This species provides a useful baseline for contextualizing venom variation within the dataset, consistent with the range of intraspecific venomic variation documented in other scorpion taxa (Newton et al., 2007; Abdel-Rahman et al., 2009; Salama and Sharshar, 2013; Schaffrath et al., 2018; Ajdi et al., 2024).

### 4.2 Venom MFPs vs DNA barcodes for species delimitation

The utility of venom MFPs as a tool for species delimitation was evaluated across multiple taxonomic scales within the monophyletic genus *Euscorpius*, encompassing different levels of genetic divergence while employing different analytical approaches and visual representations. The NMDS analyses provided a representation of relationships among deeply divergent lineages, although its resolving power decreased among closely related taxa (Fig. 9, Fig. 12). In ordination space, clusters positioned toward the right of the NMDS plot correspond to more divergent lineages, as exemplified by the outgroup, *T. flavicaudis*, and the deeply divergent *E. italicus* (Fig. 9, Fig. 12). Conversely, clusters situated near one another and/or partially overlapping correspond to closely related and morphologically similar taxa. For example, *E. latinus* and *E. trejaensis* form cohesive clusters (Fig. 9, Fig. 12) and their proximity to *E. concinnus* reflects their close relationships within the complex (Tropea, 2017; Tropea and Parmakelis, 2022). Partial overlap among these clusters, e.g., between population 43 of *E. salentinus* and the geographically adjacent population 36 of *E. garganicus* (Fig. 9, Fig. 10, Fig. 12), further suggests that geographical proximity and/or similar ecological conditions may promote convergence in venom composition despite underlying genetic divergence.

Whereas NMDS alone was unable to fully distinguish among closely related taxa, hierarchical clustering of distances in the reduced-dimensional NMDS space achieved more resolution (Fig. 10). The resulting dendrogram largely mirrored the topology of the COI phylogeny while retaining limited overlap among some heterospecific populations (Fig. 10, Fig. 14A), suggesting that evolutionary history is the primary determinant of venom composition, whereas geographical or ecological factors (e.g., environmental conditions, habitat, prey availability, and predator pressure) may modulate venom similarity at finer spatial scales (Newton et al., 2007; Abdel-Rahman et al., 2009; Pucca et al., 2014; Gangur et al., 2017). However, because these patterns are based on overall similarity metrics rather than synapomorphies, they may also reflect phenetic convergence rather than shared evolutionary history.

Although some fine-scale relationships resolved by DNA data were not recovered, the NMDS analyses were largely consistent with the COI phylogeny, indicating a high degree of concordance between venomic and genomic signals. By contrast, HCA performed directly on the intersection-based venom distance matrix successfully recovered the fine-scale structure within species that was also recovered by the COI-based phylogeny (Fig. 12). Although this approach was less effective at resolving deeper phylogenetic relationships, e.g., the erroneous placement of *E. italicus* within the *sicanus* clade and *E. aquilejensis* sister to *T. flavicaudis* (Fig. 11‒14B), it successfully discriminated among (1) closely related species within species complexes, e.g., *E. latinus* and *E. trejaensis* (Tropea and Parmakelis, 2022); (2) more divergent species within the same complex, e.g., *E. celanus* and *E. garganicus* (Podnar et al., 2021); and (3) representatives of distinct evolutionary lineages, e.g., *E. aquilejensis* and *E. italicus* (Fig. 11, Fig. 12). Similar results were reported by Zancolli et al. (2017), who used an Agilent Bioanalyzer on-chip technology-based protocol to generate a matrix of snake venom profiles and demonstrated, with NMDS analyses, that both closely related and distantly related species could be successfully discriminated, whereas closely related taxa clustered together.

It is noteworthy that the intersection-based HCA resolved *E. garganicus* into two well-supported, geographically structured, and genetically differentiated subclades (Fig. 11‒14B). Two subspecies of *E. garganicus* were previously recognized, *E. garganicus molisanus* Tropea, 2017 and the nominotypical form. Recently, Prendini (2026) elevated *E. molisanus* to the rank of species based on examination of topotypes and additional material, a decision also supported by genetic data (J. Blasco-Aróstegui and L. Prendini, unpublished data). Although both subclades recovered in the present study correspond to populations previously assigned to the nominotypical subspecies, their support by both DNA- and venom-based barcodes suggests the presence of previously unrecognized diversity in *E. garganicus*. These subclades also exhibit subtle but potentially informative morphological differences, e.g., smaller body size and distinct color patterns.

A similar pattern was observed in *E. concinnus*. Intersection-based distance HCA revealed a strong geographical signal, resolving two subclades: populations closer to the Gulf of Genoa clustered together, whereas those from Tuscany formed a separate group. However, the Tuscan group was consistently placed in an intermediate position between *E. latinus* and *E. trejaensis* across HCA analyses (Fig. 1, Fig. 11), incongruent with the relationships inferred by the DNA-based phylogeny (Fig. 12). This discordance suggests that venom compounds shared among heterospecific populations of *Euscorpius* likely reflect adaptation to similar ecological conditions known to affect venom composition independently of underlying genetic signatures (Pucca et al., 2014; Gangur et al., 2017; Schaffrath et al., 2018), e.g., habitat or diet (Newton et al., 2007; Abdel-Rahman et al., 2009; Pucca et al., 2014; Tobassum et al., 2018). Some of these populations may also represent previously unrecognized diversity caused by recent diversification processes and historical climatic events such as Pleistocene glaciations (Stefani et al., 2012; Tropea and Parmakelis, 2022). Similar taxonomic congruence between DNA phylogenies and venom profiles was reported in other venomous taxa such as cone snails, in which genetically divergent populations also presented divergent venom compositions (Puillandre et al., 2014).

Several relationships implied by the venom-based phylogeny were incongruent with morphology- and DNA-based approaches. This incongruence, together with the lack of resolution and low bootstrap values of deeper nodes (Fig. 13), may be an artefact of taxon sampling, caused by an absence of intermediate taxa that share venom components and could break up long branches by increasing the number of informative characters, which is generally expected to improve phylogenetic resolution (Kluge, 1989; Wiens, 2004).

The observed incongruence may also reflect intrinsic limitations of the venom MFP dataset, e.g., (1) homoplasy arising from mass convergence or isobarism in low-resolution MS may cause distinct peptide fragments sharing identical mass-to-charge ratios to be erroneously inferred as synapomorphies (Habler et al., 2024), and (2) potential violation of the reversibility assumption of Markovian models, as the absence of a peptide peak may not correspond to the loss of a toxin-coding locus, but may instead result from differential gene expression, post-translational processing, proteolytic cleavage, or analytical detection limits (Calvete, 2014; Modahl et al., 2020).

Two limitations of the approach applied in the present study should be addressed in future studies. Filtering MFPs to retain highly specific components, together with the absence of intermediate taxa bridging deeply divergent lineages, e.g., among *E. aquilejensis*, *E. italicus*, and *E. celanus*, may have obscured potentially synapomorphic peaks and thereby reduced resolution at deeper phylogenetic levels, as suggested by the ML phylogeny. Broader taxon sampling should improve the resolution of venom-based species delimitation and increase congruence with genetic and morphological datasets by increasing the number of phylogenetically informative characters as demonstrated in a study of snake phylogeny using lipids as characters (Kluge, 1989).

### 4.3 Future directions

The present study aimed to provide a one-to-one comparison between genetic and venomic data across multiple Italian species of the scorpion genus *Euscorpius*, a monophyletic group. Inspired by early efforts to detect species-specific signals in venomes, particularly using MFP data, with the aim of distinguishing species based on venom profiles (Dyason et al., 2002; Schaffrath, 2017; Schaffrath et al., 2018; Ajdi et al., 2024), the sampling design included closely related and more divergent taxa, enabling an explicit assessment of the congruence between DNA-based and venom-based barcoding approaches and their relative efficacy for species delimitation.

Based on the study of Italian *Euscorpius*, venom MFPs showed considerable potential for species delimitation using similarity-based approaches and may be used to augment other sources of data for resolving cryptic species complexes, morphologically similar or genetically conserved taxa, as well as for cases in which different sources of data provide conflicting signals. Examples include the scorpion genera *Alpiscorpius* Gantenbein et al., 1999 and *Hadogenes* Kraepelin, 1894, within which species with little or no morphological differentiation and low genetic divergence have been delimited partially or entirely by cytogenetic evidence (e.g., karyotype races; Newlands and Cantrell, 1985; Prendini, 2001; Kovařík et al., 2019; Štundlová et al., 2019).

Another promising avenue for the use of venom MFPs concerns the development of reference databases of taxon-specific combinations of ion signals. In principle, such diagnostic profiles could allow the assignment of unidentified specimens to known taxa based on MFPs, conceptually analogous to DNA barcoding (Schaffrath, 2017). Currently, this application is contingent on substantially expanded and taxonomically balanced sampling to ensure that venomic diversity is adequately represented across lineages. Beyond taxonomy, comprehensive venom databases would also provide resources for comparative studies and facilitate identification of bioactive compounds with potential biomedical and pharmacological applications, e.g., ScorpDb (Baradaran et al., 2024).

In conclusion, although venom MFPs appear highly effective for species discrimination across various levels of relatedness, DNA-based approaches currently provide higher resolution with limited taxon sampling. Venom MFPs may achieve comparable discriminatory power with broader taxon sampling and methodological optimization.

## CRediT authorship contribution statement

**Javier Blasco-Aróstegui:** Writing – review & editing, Writing – original draft, Visualization, Software, Project administration, Methodology, Investigation, Funding acquisition, Formal analysis, Data curation, Conceptualization. **Massimiliano Roppo:** Writing – review & editing, Validation, Methodology, Investigation, Data curation. **Lorenzo Prendini:** Writing – review & editing, Visualization, Supervision, Resources, Project administration, Funding acquisition, Data curation, Conceptualization. **Reinhard Predel:** Writing – review & editing, Visualization, Validation, Supervision, Resources, Project administration, Funding acquisition, Data curation, Conceptualization.

## Funding

J.B.-A. was supported by a doctoral fellowship from the Fundação para a Ciência e a Tecnologia, Portugal (https://doi.org/10.54499/2021.04967.BD). Fieldwork was supported by an Exploration Fund Grant (2023) from the 10.13039/100002861Explorers Club to J.B.-A., a grant from the Vincent Roth Fund for Systematics Research (2022) of the 10.13039/100016472American Arachnological Society awarded to J.B.-A., and NSF grants DEB-1655050 and DEB-2003382 to L.P. The 10.13039/100005835AMNH OTPS budget of L.P. covered DNA sequencing, whereas the OTPS funds of R.P. from the Institute for Zoology, 10.13039/501100008001University of Cologne (UC), supported the generation of venom mass fingerprints and the materials for venom collection during fieldwork, as well as lodging for J.B.-A. and M.R. in Cologne, Germany.

## Declaration of competing interest

The authors declare that we have no competing financial interests that could have influenced the work reported in this manuscript.

## Data Availability

All raw mass spectrometry data were deposited at the ProteomeXchange Consortium via the Proteomics Identification Database (PRIDE) of the European Bioinformatics Institute (EMBL-EBI, accesion code PXD084418; https://www.ebi.ac.uk/pride/); and genetic data deposited in GenBank (https://www.ncbi.nlm.nih.gov/genbank) with accession codes for individual sequences shown in Table S2.
