# Supplementary material for: Venom mass fingerprints for species delimitation, reloaded: A test case with Italian scorpions (Euscorpius)
Source: Toxicon X. 2026 Sep 21;32:100265. doi: 10.1016/j.toxcx.2026.100265 (PMC13677054; doi:10.1016/j.toxcx.2026.100265)
Supplement: Supplementary file 1 [file mmc1.docx]

**Venom Mass Fingerprints for Species Delimitation, Reloaded: A Test Case with Italian Scorpions (*Euscorpius*)**

**Javier Blasco-Aróstegui^1,2,3^, Massimiliano Roppo^2,3^, Lorenzo Prendini^2^ & Reinhard Predel^3^**

^1^*Centre for Ecology, Evolution and Environmental Changes, Departamento de Biologia Animal, Faculdade de Ciências, Universidade de Lisboa, Campo Grande 016, 1749-016 Lisbon, Portugal;* email: [javierblasco77@gmail.com](mailto:javierblasco77@gmail.com) ORCID: <https://orcid.org/0000-0003-4230-9375>

^2^*Arachnology Lab and Scorpion Systematics Research Group, Division of Invertebrate Zoology, American Museum of Natural History;* email: [mroppo@outlook.it](mailto:mroppo@outlook.it), [lorenzo@amnh.org](mailto:lorenzo@amnh.org) ORCID: <https://orcid.org/0009-0008-9816-7373>, <https://orcid.org/0000-0001-8727-7106>

^3^*Institute of Zoology, University of Cologne, Zülpicher Str. 47b, 50674 Köln, Germany, University of Cologne, Köln, Germany*; email: [reinhard.predel@uni-koeln.de](mailto:reinhard.predel@uni-koeln.de) ORCID: <https://orcid.org/0000-0002-0202-6672>

**Supplementary Information**

**Table S1 …………………..………………………………………………………….………. 2**

**Table S2 …………………..………………………………………………………….……..... 3**

**Table S3 …………………..………………………………………………………….…….... 5 Table S4 …………………..………………………………………………………….……..... 6**

**Figure S1 …………………..…………………………………………………………………. 7**

**Figure S2 …………………..………………………………………………………………..... 8**

**Figure S3 …………………..………………………………………………………………..... 9**

**Figure S4 …………………..………………………………………………………………... 10**

**Table S1.** Binary presence/absence matrix used in Non-metric Multidimensional Scaling and Hierarchical Clustering Analyses, including all filtered peaks for 52 populations representing eight Italian species of *Euscorpius* Thorell, 1876, and *Tetratrichobothrius flavicaudis* (DeGeer, 1778). Between 20 and 31 of most intense and consistently detected venom molecular masses selected per individual, for intra- and interspecific comparisons. Peak numbers (1‒304) at top of each column, corresponding molecular mass values (*m/z*) at bottom (3017‒9025). Abbreviations: Sp., Species; Pop., Population; MR, Venom sample code; *aqu*, *Euscorpius aquilejensis* (C.L., Koch, 1837); *cel*, *Euscorpius celanus* Tropea, 2012; *con*, *Euscorpius concinnus* (C.L. Koch, 1837); *gar*, *Euscorpius garganicus* Caporiacco, 1950; *lat*, *Euscorpius latinus* Tropea & Parmakelis, 2022; *sal*, *Euscorpius salentinus* Tropea, 2017; *tre*, *Euscorpius trejaensis* Tropea & Parmakelis, 2022; *fla*, *T. flavicaudis*.

(Table S1 Excel file)

**Table S2.** Tissue samples and Genbank accession codes for newly generated DNA sequences of Cytochrome *c* Oxidase Subunit I (COI) loci used in phylogenetic analyses of eight Italian species of *Euscorpius* Thorell, 1876 and *Tetratrichobothrius flavicaudis* (DeGeer, 1778). Tissue samples deposited in Ambrose Monell Cryocollection (AMCC) at American Museum of Natural History, New York.

| **Species** | **AMCC** | **Locality** | **Georeference** | **COI** |
| --- | --- | --- | --- | --- |
| *Tetratrichobothrius flavicaudis* | LP 18791 | Roma | 41°55.594'N 12°29.931'E | QB031148 |
|  | LP 18821 | Norma | 41°34.211'N 13°00.499'E | QB031147 |
|  | LP 19019 | Roma | 41°55.8013'N 12°29.9942'E | QB031150 |
|  | LP 19020 | Roma | 41°54.8215'N 12°29.0878'E | QB031149 |
| *Euscorpius aquilejensis* | LP 18896 | Ponte Chiaradovo | 43°25.53'N 12°59.669'E | QB031103 |
|  | LP 18907 | Caramanico Terme | 42°09.425'N 14°00.701'E | QB031100 |
|  | LP 18913 | Gole di Aielli-Celano | 42°05.306'N 13°34.126'E | QB031101 |
|  | LP 19018 | Isola San Domino | 42°06.643'N 15°28.801'E | QB031102 |
|  | LP 19023 | Aventino | 41°53.162'N 12°28.98'E | QB031104 |
| *Euscorpius celanus* | LP 18801 | Gole di Aielli-Celano | 42°05.102'N 13°34.038'E | QB031105 |
|  | LP 18805 | Gole di Aielli-Celano | 42°05.306'N 13°34.126'E | QB031106 |
|  | LP 18875 | Teramo | 42°41.601'N 13°39.815'E | QB031108 |
|  | LP 18900 | Castel del Monte | 42°22.05'N 13°43.45'E | QB031109 |
|  | LP 18910 | Gole di Aielli-Celano | 42°05.306'N 13°34.126'E | QB031107 |
| *Euscorpius concinnus* | LP 18824 | Ponte a Bozzone | 43°21.0200'N 11°23.1683'E | QB031111 |
|  | LP 18829 | Gaiole in Chianti | 43°28.106'N 11°26.185'E | QB031112 |
|  | LP 18837 | Montaione | 43°32.647'N 10°54.919'E | QB031113 |
|  | LP 18838 | Fortezza di Montealfonso | 44°06.593'N 10°24.115'E | QB031114 |
|  | LP 18843 | Resceto | 44°05.053'N 10°12.414'E | QB031115 |
|  | LP 18848 | Signora di Montallegro | 44°22.251'N 09°15.337'E | QB031116 |
|  | LP 18852 | Gambatesa Mine | 44°21.45'N 09°27'E | QB031117 |
|  | LP 18854 | Punta Mesco | 44°08.644'N 09°38.245'E | QB031118 |
|  | LP 18862 | Pignone | 44°10.473'N 09°42.918'E | QB031119 |
|  | LP 18863 | Volastra | 44°07.085'N 09°44.423'E | QB031120 |
|  | LP 18872 | Barberino del Mugello | 44°00.667'N 11°13.422'E | QB031121 |
| *Euscorpius garganicus* | LP 18920 | Montefalcone nel Sannio | 41°51.73'N 14°38.657'E | QB031122 |
|  | LP 18926 | Gambatesa | 41°30.129'N 14°54.673'E | QB031123 |
|  | LP 18927 | Macchia Valforte | 41°35.505'N 14°54.53'E | QB031124 |

**Table S2.** Continued.

| **Species** | **AMCC** | **Locality** | **Georeference** | **COI** |
| --- | --- | --- | --- | --- |
| *Euscorpius garganicus* | LP 18944 | San Marco in Lamis | 41°42.891'N 15°40.133'E | QB031125 |
|  | LP 18946 | Massafra | 40°36.172'N 17°06.839'E | QB031129 |
|  | LP 18952 | Massafra | 40°36.225'N 17°06.979'E | QB031130 |
|  | LP 18961 | Forest Gallioli Cognato | 40°30.381'N 16°09.223'E | QB031131 |
|  | LP 18964 | Stigliano | 40°22.455'N 16°19.351'E | QB031132 |
|  | LP 18969 | Policoro | 40°11.147'N 16°42.398'E | QB031133 |
|  | LP 18984 | Castro | 40°00.146'N 18°25.26'E | QB031151 |
|  | LP 19000 | Lucito | 41°43.72'N 14°41.405'E | QB031126 |
|  | LP 19012 | Isola San Domino | 42°06.779'N 15°29.044'E | QB031127 |
|  | LP 19017 | Isola San Domino | 42°06.643'N 15°28.801'E | QB031128 |
| *Euscorpius italicus* | LP 18793 | Calcata | 42°13.041'N 12°25.221'E | QB031134 |
| *Euscorpius latinus* | LP 18811 | Anticolli Corrado | 42°00.776'N 12°58.248'E | QB031135 |
|  | LP 18812 | Lucretili Mts. | 42°05.529'N 12°53.008'E | QB031136 |
|  | LP 18818 | Norma | 41°33.857'N 13°00.957'E | QB031110 |
|  | LP 18884 | Castel Fusano | 41°42.989'N 12°19.261'E | QB031137 |
|  | LP 18917 | Villa San Michele | 41°44.710'N 14°13.978'E | QB031138 |
| *Euscorpius salentinus* | LP 18974 | Otranto | 40°12.393'N 18°27.253'E | QB031139 |
|  | LP 18980 | Melendugno | 40°14.172'N 18°23.78'E | QB031140 |
|  | LP 18990 | Diso | 40°00.331'N 18°23.914'E | QB031141 |
|  | LP 18994 | Vignacastrisi | 40°00.728'N 18°24.954'E | QB031142 |
|  | LP 18997 | Brenca | 41°01.79'N 17°03.2'E | QB031143 |
| *Euscorpius trejaensis* | LP 18800 | Calcata | 42°13.1718'N 12°25.0500'E | QB031144 |
|  | LP 18889 | Monti della Tolfa | 42°04.551'N 11°57.776'E | QB031145 |
|  | LP 18890 | Castel Giuliano | 42°03.005'N 12°07.488'E | QB031146 |

**Table S3.** Intraspecific venom variation of 53 mass fingerprints from eight Italian species of *Euscorpius* Thorell, 1876 and *Tetratrichobothrius flavicaudis* (DeGeer, 1778), based on group dispersions and mean distances from centroid in PERMDISP analysis.

| **Species** | **Mean Distance to Centroid** | **Standard Deviation** |
| --- | --- | --- |
| *E. aquilejensis* | 0.0793 | 0.0562 |
| *E. celanus* | 0.4018 | 0.1858 |
| *E. concinnus* | 0.3918 | 0.1705 |
| *E. garganicus* | 0.4906 | 0.0860 |
| *E. italicus* | 0.1429 | 0.0000 |
| *E. latinus* | 0.3722 | 0.1312 |
| *E. salentinus* | 0.1288 | 0.1447 |
| *E. trejaensis* | 0.2772 | 0.1116 |
| *T. flavicaudis* | 0.3439 | 0.0921 |

**Table S4.** Mean interspecific and intraspecific (bold) uncorrected pairwise distances of Cytochrome *c* Oxidase Subunit I (COI) locus for eight Italian species of *Euscorpius* Thorell, 1876 and *Tetratrichobothrius flavicaudis* (DeGeer, 1778). Abbreviations: *aqu*, *Euscorpius aquilejensis* (C.L., Koch, 1837); *cel*, *Euscorpius celanus* Tropea, 2012; *con*, *Euscorpius concinnus* (C.L. Koch, 1837); *gar*, *Euscorpius garganicus* Caporiacco, 1950; *lat*, *Euscorpius latinus* Tropea & Parmakelis, 2022; *sal*, *Euscorpius salentinus* Tropea, 2017; *tre*, *Euscorpius trejaensis* Tropea & Parmakelis, 2022; *fla*, *Tetratrichobothrius flavicaudis* (DeGeer, 1778).

|  | *aqu* | *cel* | *con* | *gar* | *ita* | *lat* | *sal* | *tre* | *fla* |
| --- | --- | --- | --- | --- | --- | --- | --- | --- | --- |
| *aqu* | **0.0077** |  |  |  |  |  |  |  |  |
| *cel* | 0.0924 | **0.0075** |  |  |  |  |  |  |  |
| *con* | 0.0849 | 0.0859 | **0.0336** |  |  |  |  |  |  |
| *gar* | 0.1004 | 0.0801 | 0.0888 | **0.052** |  |  |  |  |  |
| *ita* | 0.0894 | 0.0859 | 0.0813 | 0.0943 | **0.0037** |  |  |  |  |
| *lat* | 0.0812 | 0.0850 | 0.0433 | 0.0895 | 0.0842 | **0.0059** |  |  |  |
| *sal* | 0.0900 | 0.0891 | 0.0944 | 0.0976 | 0.0897 | 0.0981 | **0.0056** |  |  |
| *tre* | 0.0905 | 0.0830 | 0.0475 | 0.0882 | 0.0905 | 0.0336 | 0.0973 | **0.0019** |  |
| *fla* | 0.1275 | 0.1219 | 0.1265 | 0.1314 | 0.1136 | 0.1209 | 0.1289 | 0.1269 | **0.0529** |

**Figure S1.** Venom mass fingerprints (MFPs) of three specimens of *Tetratrichobothrius flavicaudis* (DeGeer, 1778) showing little variation in venom composition following regeneration. Freshly regenerated venom collected 24 hours after initial stimulation and venom depletion compared with samples obtained after longer recovery periods. **A.** Specimen 3353, 24 h post-initial extraction. **B.** Specimen 3353, 72 h post-initial extraction. **C.** Specimen 3354, 24 h post-initial extraction. **D.** Specimen 3354, 7 days post-initial extraction. **E.** Specimen 3350, 24 h post-initial extraction. **F.** Specimen 3350, 5 days post-initial extraction.

**Figure S2.** Comparison of Non-metric Multidimensional Scaling ordinations based on three distance measures: Bray-Curtis, Dice, and Jaccard. Ordinations derived from all indices yielded identical stress values (0.1219). Bray-Curtis and Jaccard yielded identical ordination configurations.

**Figure S3.** Dispersion boxplots showing mean distance to centroid for 53 venom mass fingerprints from eight Italian species of *Euscorpius* Thorell, 1876 and *Tetratrichobothrius flavicaudis* (DeGeer, 1778), as estimated by PERMDISP.

**Figure S4.** Cophenetic correlation of eleven distance measures used in Hierarchichal Clustering Analyses with Ward’s method of 52 venom mass fingerprints from eight Italian species of *Euscorpius* Thorell, 1876 and *Tetratrichobothrius flavicaudis* (DeGeer, 1778). Higher cophenetic correlation indicates better representation of distances.
